# Fluctuating and Stable High Temperatures Differentially Affect Reproductive Endocrinology of Female Pupfish

**DOI:** 10.1093/iob/obae003

**Published:** 2024-02-01

**Authors:** M J Housh, J Telish, K L Forsgren, S C Lema

**Affiliations:** Biological Sciences Department, Center for Coastal Marine Sciences, California Polytechnic State University, San Luis Obispo, CA 93407, USA; Department of Biological Science, California State University, Fullerton, CA 92834, USA; Department of Biological Science, California State University, Fullerton, CA 92834, USA; Biological Sciences Department, Center for Coastal Marine Sciences, California Polytechnic State University, San Luis Obispo, CA 93407, USA

## Abstract

For many fishes, reproductive function is thermally constrained such that exposure to temperatures above some upper threshold has detrimental effects on gametic development and maturation, spawning frequency, and mating behavior. Such impairment of reproductive performance at elevated temperatures involves changes to hypothalamic–pituitary–gonadal (HPG) axis signaling and diminished gonadal steroidogenesis. However, how HPG pathways respond to consistently high versus temporally elevated temperatures is not clear. Here, sexually mature Amargosa River Pupfish (*Cyprinodon nevadensis amargosae*) were maintained under thermal regimes of either stable ∼25°C (low temperature), diurnal cycling temperatures between ∼27 and 35°C (fluctuating temperature), or stable ∼35°C (high temperature) conditions for 50 days to examine effects of these conditions on HPG endocrine signaling components in the pituitary gland and gonad, ovarian and testicular gametogenesis status, and liver gene expression relating to oogenesis. Female pupfish maintained under stable high and fluctuating temperature treatments showed reduced gonadosomatic index values as well as a lower proportion of oocytes in the lipid droplet and vitellogenic stages. Females in both fluctuating and stable 35°C conditions exhibited reduced ovarian mRNAs for steroid acute regulatory protein (*star*), cholesterol side chain-cleavage enzyme, P450scc (*cyp11a1*), and 3β-hydroxysteroid dehydrogenase (*3bhsd*), while ovarian transcripts encoding 11β-hydroxysteroid dehydrogenase (*11bhsd*) and sex hormone-binding globulin (*shbg*) were elevated in females at constant 35°C only. Ovarian aromatase (*cyp19a1a*) mRNA levels were unaffected, but circulating 17β-estradiol (E_2_) was lower in females at 35°C compared to the fluctuating temperature condition. In the liver, mRNA levels for choriogenins and vitellogenin were downregulated in both the fluctuating and 35°C conditions, while hepatic estrogen receptor 2a (*esr2a*) and *shbg* mRNAs were elevated in 35°C females. Taken together, these results demonstrate the potential for elevated temperatures to impair ovarian steroidogenesis and reduce egg envelope and vitellogenin protein production in female *C. n. amargosae* pupfish, while also shedding light on how thermal regimes that only intermittently reach the upper thermal range for reproduction have differential impacts on reproductive endocrine pathways than constantly warm conditions.

## Introduction

Reproduction in fishes occurs over a limited range of temperatures, with that range varying widely depending on the latitude, geographic range, and specific thermal conditions under which the taxon evolved (Sumpter 1990; Pankhurst and Porter 2003). Across fishes, exposure to unusually high or prolonged elevations in temperature can negatively impact reproduction, typically through reduced gamete production, gamete quality, and embryo viability (Van Der Kraak and Pankhurst 1997; Miranda et al. 2013; Alix et al. 2020; Zahangir et al. 2022; Lema et al. 2024). Such effects on reproduction occur, in part, via functional changes in the hypothalamic–pituitary–gonadal (HPG) endocrine axis, which regulates several reproductive processes, including gametogenesis, gamete maturation, and spawning behaviors (Sumpter 1990; Pankhurst and Porter 2003; Pankhurst and Munday 2011; Servili et al. 2020). Elucidating the mechanisms by which elevated temperature impairs reproductive function in fishes is imperative for forecasting how vulnerable populations will fare under current and future climate warming (O'Reilly et al. 2015; Frölicher and Laufkötter 2018) as well as determining the potential for physiological acclimation or adaptation to warmer environments (Pörtner and Peck 2010).

As in other vertebrates, the HPG axis in teleosts plays a fundamental role in regulating sex steroid hormone production and reproduction in both sexes. Under environmental conditions favorable for reproduction, the hypothalamus initiates a signaling cascade of hormones that proceeds through the pituitary gland to the gonads, culminating in the development and, ultimately, spawning of gametes (Van Der Kraak and Pankhurst 1997; Pankhurst and Munday 2011; Yaron and Levavi-Sivan 2011; Servili et al. 2020). Hypothalamic neuroendocrine signaling molecules, including gonadotropin-releasing hormone I (Gnrh-I), are released directly from neuronal axons into the hypophysis of fish, prompting the pituitary production and secretion of two gonadotropins (GtH): follicle-stimulating hormone (Fsh) and luteinizing hormone (Lh). Once bound to their respective receptors, these GtHs stimulate the gonadal production of sex steroids that perform different roles based on sex. Ovarian production of 17β-estradiol (E_2_) promotes oogonial proliferation and vitellogenesis, while in the testes, 11-ketotestosterone (11-KT) is the primary androgen regulating spermatogenesis and spermiogenesis (Nagahama 1994; Swanson et al. 2003).

In many fish species examined to date, the development and maturation of oocytes appear to be particularly vulnerable to deleterious effects of abnormally high temperatures (Alix et al. 2020; Lema et al. 2024). Exposure to elevated temperatures during oogenesis can have repercussions for egg development, the timing of egg maturation, and egg size and composition, which together can have impacts for fecundity, the timing of spawning, and offspring viability (Elliott 1981; Tveiten and Johnsen 2001; Pankhurst and Munday 2011; Miranda et al. 2013; Lema et al. 2024; reviewed in Alix et al. 2020). While rising temperatures within a species’ normal range of thermal conditions can initially promote or even accelerate oocyte growth, beyond a certain threshold, high temperatures often have negative consequences for oogenesis (reviewed in Alix et al. 2020; Lema et al. 2024). For example, sexually mature female rainbow trout (*Oncorhynchus mykiss*) maintained at temperatures from 9 to 21°C showed clear patterns of temperature-dependent effects on egg production and survival, with fish at the higher 18–21°C conditions exhibiting low production and complete egg mortality (Pankhurst et al. 1996). Adult female sheepshead minnow (*Cyprinodon variegatus*) maintained at 37°C for 14 day had lower gonadosomatic index (GSI) values compared to those held at 27°C (Bock et al. 2021), a temperature in the optimal range for reproduction in this eurythermal species. Female sheepshead minnow at 37°C also had lower concentrations of circulating E_2_ and significantly fewer spawning capable oocytes in the ovary (Bock et al. 2021). And, in the Atlantic wolffish (*Anarhichas lupus*), a coldwater stenothermal species, exposure to elevated temperatures during vitellogenesis delayed ovulation and reduced egg production per female (Tveiten and Johnsen 1999). In a follow-up study, Tveiten and Johnsen (2001) reported that the inhibitory effects of elevated temperature were underlain by changes in the timing of plasma T and E_2_ peak concentrations during oogenesis.

Based on those and other studies, it has become clear that exposure of fish to temperatures near or exceeding the upper limits of their reproductive range alters the production and action of hormones within reproductive endocrine pathways, thereby disrupting gametogenesis (Alix et al. 2020; Servili et al. 2020). For instance, in red seabream (*Pagrus major*), a warmer water temperature of 25°C suppressed both the development of oocytes in ovarian portions of hermaphroditic gonads as well as gonadal mRNA expression of the steroidogenic enzymes cytochrome P450 aromatase (*cyp19a1a*) and 11β-hydroxylase (*11bhsd*) relative to fish maintained at 15 or 20°C (Lim et al. 2003). Similarly, adult pejerrey (*Odontesthes bonariensis*) maintained at elevated temperature of 27°C showed atretic oocytes in the ovary, lower plasma E_2_, and reduced relative mRNA levels for luteinizing hormone β-subunit (*lhβ*) in the pituitary gland and follicle-stimulating hormone receptor (*fshr*) in the ovary, implying alterations at multiple levels of HPG axis signaling (Soria et al. 2008).

While these studies are providing a picture of the general mechanisms by which exposure to atypically high or extended elevations in temperature affect reproductive physiology and performance in fishes, the experimental treatments used in such studies do not always represent natural conditions that fish may experience in the wild. To date, most studies examining temperature effects on reproductive endocrinology have used constant, or stable, temperature conditions (see review by Alix et al. 2020), and yet many fishes, including inland species of riverine and lacustrine habitats—and marine species occupying dynamic habitats such as estuaries or tidepools—experience thermally variable conditions. Thermally variable conditions affect the physiology and behavior of fish in ways that are different than stable or chronic thermal stress (Morash et al. 2021). Physiological states of fish measured under stable temperature conditions have been observed to vary from those of fish under fluctuating thermal conditions, even with the same average temperature (e.g., Meeuwig et al. 2004; Imholt et al. 2011; Beauregard et al. 2013). For instance, fish maintained under fluctuating temperature conditions have been found to have dissimilar profiles of hepatic gene expression (Podrabsky and Somero 2004; Logan and Buckley 2015), metabolic rate (Beauregard et al. 2013), and growth rate (Meeuwig et al. 2004; Imholt et al. 2011; Recsetar et al. 2014). Comparatively few studies, however, have examined the influences of fluctuating temperatures on reproductive endocrinology of fishes, despite such variable thermal regimes being ecologically relevant to many species. Turquoise killifish (*Nothobranchius furzeri*) maintained under a thermal regime that fluctuated daily from 20 to 30°C were smaller in body size but had higher fecundity and a longer mean lifespan than conspecifics under a stable 27.5°C (Žák and Reichard 2020). And, in one study with pejerrey, Elisio et al. (2012) found that fish exposed to a daily fluctuating regime of elevated range (19–27°C) ceased spawning, showed gonadal regression, and downregulated HPG axis signaling relative to fish under a lower fluctuating range (17–19°C). Based on those observations, it is clear that fluctuating temperatures can affect reproductive performance in fishes. However, it remains unclear whether exposure of fish to fluctuating thermal conditions only reaching stressful temperatures intermittently affects similar reproductive endocrine signaling pathways as chronic (stable) exposures or whether endocrine signaling recovery might occur during the period when temperature drops below a threshold for detrimental impacts in thermal regimes with large diurnal variation.

In this study, we examined the effects of different temperature regimes on the reproductive endocrinology of adult Amargosa River pupfish (*C. nevadensis amargosae*) to better understand changes in HPG axis-associated signaling and tissues that occur with high temperature exposure, and, to evaluate whether the effects of elevated temperatures on those pathways differ when fish experience fluctuating thermal conditions compared to chronic (stable) high temperature conditions. Like other pupfishes in the southwestern region of the United States and northern Mexico, the Amargosa River pupfish has evolved one of the broadest and highest thermal tolerances of any fish (Feldmeth 1981; Minckley and Minckley 1986; Heath et al. 1993; Bennett and Beitinger 1997; Beitinger et al. 2000; Nordlie 2006; Carson et al. 2014), and a conspecific population of subspecies *C. n. nevadensis* has the highest experimentally documented temperature for reproduction of any fish recorded to date (Shrode and Gerking 1977; see Motani and Wainwright 2015). The Amargosa River pupfish lives primarily within the Amargosa River, a desert stream environment that experiences extreme temperature fluctuations both daily (over 25°C fluctuations between day and night) and seasonally (varying from near freezing to over 40°C) (Lema et al. 2019). In this study, mixed-sex groups of sexually mature *C. n. amargosae* were maintained under three temperature treatments: (1) a stable lower temperature condition held constant at 25°C (*Low*), (2) a fluctuating (*Fluctuating*) condition that oscillated daily from 25°C at 09:00 to a high temperature of 35°C at 16:00, and (3) a stable high temperature condition held constant at 35°C (*High*). The *Low* treatment represented chronic exposure conditions thought to be near optimal for reproduction (27–28°C). The *High* condition was at the demonstrated upper end for reproduction (36°C) for *C. n. nevadensis* in captivity (Shrode and Gerking 1977) and the observed upper limit for spawning by *C. n. amargosae* in their natural river habitat (Soltz 1974). That 35°C *High* temperature was also similar to conditions used in two recent studies of high temperature effects on reproductive endocrinology in *Cyprinodon* pupfishes (Bock et al. 2021; Lema et al. 2022). The *Fluctuating* treatment was selected to be more representative of the temperature variation experienced by these fish in their natural habitat over a 24 h cycle (Lema et al. 2019). Adult female and male *C. n. amargosae* pupfish were maintained under those *Low, Fluctuating*, or *High* thermal regimes for 50 day after which several indices of reproductive status and endocrine regulation were measured, including circulating E_2_ in females and 11-KT in males, relative gene transcripts encoding gonadotropins in the pituitary gland and steroidogenic enzymes in the gonad tissues of both sexes, and vitellogenin and choriogenin proteins in the livers of female pupfish. In addition, hepatic and gonadal mRNA levels of sex hormone-binding globulin (*shbg*), which binds and modulates the free hormone concentration of sex steroids in the blood (Marivin et al. 2014; Laurent et al. 2016), were also quantified.

## Materials and methods

### Animal collection and experimental temperature treatments

On October 18, 2020, adult *C. n. amargosae* pupfish were collected from the Amargosa River (35°51.275′N, 116°13.833′W) located in the Death Valley region of southeastern California, USA. Fish were collected using minnow traps and transported to [redacted] University, where they were maintained in 208 L tanks with recirculating dechlorinated water (API® Tap Water Conditioner) at <0.4 ppt and room temperature (∼24–25°C) to allow for acclimation to captive conditions.

After 2 months of acclimation to captivity, fish were transferred to 113 L experimental treatment tanks with six males and six females per tank and three replicate tanks per temperature treatment condition, for a total of *n* = 18 females and 18 males per treatment. However, two male fish were misidentified as females prior to experimentation resulting in *n* = 19 males for two of the three treatments. Each fish was weighed (males: 3.35 ± 1.32 g [mean ± standard deviation]; females: 2.47 ± 0.94 g) and measured (standard length [SL]; males: 43.42 ± 5.46 mm; females: 40.13 ± 4.67 mm), and then assigned to an experimental tank systematically to balance body size variation for male and female fish, separately, across the tanks, resulting in similar distributions of body mass and length for both males or females across treatments (one-factor analysis of variance [ANOVA] models, *P* ≥ 0.885). The mixed-sex groups of pupfish were then acclimated in the experimental tanks under room temperature conditions (24.0 ± 0.86°C) (mean ± standard deviation) for ∼3 months prior to beginning exposure of the fish to the different experimental temperatures.

**Fig. 1:**
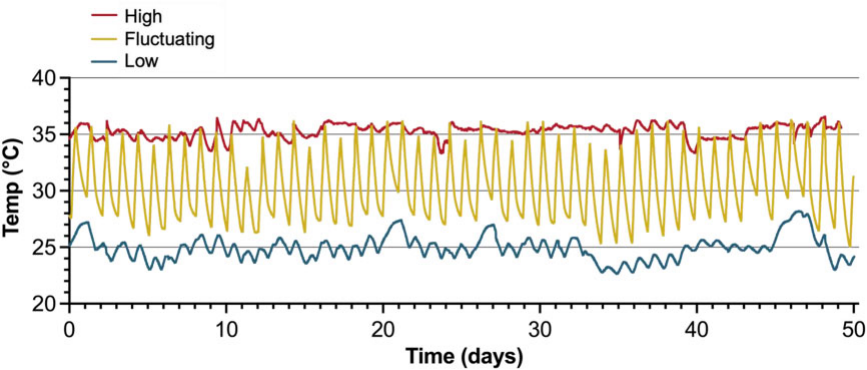
Profiles of the three temperature treatments over 50 days of experimental conditioning. “*Low*” was held at 24.92 ± 0.68°C (mean ± SD), “*Fluctuating*,” varied 8.08 ± 1.02°C daily between a minimum temperature of 27.17 ± 1.05°C at 09:00 and a maximum temperature of 35.30 ± 0.59°C at 16:00, with an overall mean temperature of 29.52 ± 2.46°C, and “*High*” was held at 35.09 ± 0.70°C.

Experimental temperature treatments consisted of three conditions: a stable low (*Low*) temperature condition (∼25°C), a fluctuating (*Fluctuating*) temperature condition that cyclically fluctuated each day from ∼27 to 35°C, and a stable high (*High*) temperature condition (∼35°C). The fluctuating and high temperature treatment tanks were heated using Hygger HG-802 aquarium heaters (Shenzhen Mago Co., Ltd, Shenzhen City, Guangdong Province, China). Temperatures of the three experimental systems were logged every 10 min (HOBO U12-008 4-Channel External Data Logger; Onset Computer Corp., MA, USA). Recorded temperature profiles over the experimental exposure period were as follows (Fig. 1): (1) *Low* treatment: 24.92 ± 0.68°C (mean ± standard deviation), (2) *Fluctuating* treatment: temperature fluctuated 8.08 ± 1.02°C daily between a minimum of 27.17 ± 1.05°C at 09:00 h and a maximum of 35.30 ± 0.59°C at 16:00 h, with an overall mean temperature of 29.52 ± 2.46°C, and (3) *High* treatment: 35.09 ± 0.70°C. Throughout the experimental period, fish were maintained in fresh (salinity 0.2 ppt) dechlorinated tap water (<0.4 ppt) under a 16L:8D photoperiod and fed dried spirulina flake feed (Aquatic Eco-Systems, Inc., Apopka, FL, USA) *ad libitum* once or twice daily.

### Tissue and blood sampling

Fish were sampled after 49 or 50 day of exposure to the experimental temperature treatments, with sampling balanced across treatment groups on the consecutive sampling days. All fish were sampled between the hours of 11:00 and 19:00. The *High* treatment group experienced more mortalities than *Low* or *Fluctuating* during experimental conditioning, resulting in the following sampling numbers: *n* = 17 females and *n* = 19 males from the *Low* temperature treatment, *n* = 15 females and *n* = 19 males from the *Fluctuating* treatment, and *n* = 10 females and *n* = 10 males from the *High* treatment.

Fish were individually netted and euthanized in an aqueous solution of tricaine methanesulfonate (MS222; Argent Chemicals, Redmond, WA, USA). Total mass and SL were recorded, and blood was collected in heparinized capillary tubes from the caudal vasculature of each fish and then centrifuged at 3000 × *g* 10 min at 4°C to isolate plasma. Plasma samples were stored at −70°C until subsequent hormone quantification. The hypothalamus and pituitary gland were dissected and separately flash frozen in liquid N_2_ before storage at −70°C. The liver and gonads were dissected and weighed for determination of GSI and hepatosomatic index (HSI) values, which were calculated using the formula [Organ Weight/Total Body Weight] × 100. Liver and gonad tissues were each divided; all apportioned liver samples were flash frozen in liquid N_2_, while one part of the divided gonad was flash frozen in liquid N_2_ for gene expression analyses and the other part was fixed in a solution of 4% paraformaldehyde in phosphate-buffered saline (PBS) buffer for 24 h at 4°C, followed by a solution of 4% paraformaldehyde and 30% sucrose in PBS, and then 30% sucrose in PBS until embedding for histological analysis. Multiple factors contributed to a reduced number of male testes samples from the *High* treatment group used in the analyses of male GSI and testicular gene expression, including fish mortality, samples lost to liquid N_2_ intrusion into the RNase/DNase-free microcentrifuge tube, or an insufficient RNA amount.

### Quantification of plasma E_2_ and 11-KT

Plasma concentrations of E_2_ and 11-KT were quantified using enzyme-linked immunosorbent assays (ELISA) (Cayman Chemical, Inc., Ann Arbor, MI, USA). Due to the limited amount of plasma obtained from individual *C. n. amargosae* fish, E_2_ was assayed in females only, and 11-KT was assayed in males only. For the E_2_ ELISA, 4 μL of plasma from female pupfish was incubated for 2 h at room temperature with E_2_ acetylcholinesterase (AChE) tracer and E_2_ ELISA antiserum in a 96-well plate pre-coated with mouse monoclonal anti-rabbit IgG. After incubation, the plate was developed with Ellman's reagent for 60 min and then read at 405 nm on a Victor X4 Microplate Reader (PerkinElmer, Waltham, MA, USA). The E_2_ ELISA standard curve spanned from 0.61 to 10,000 pg mL^−1^ E_2_. For the 11-KT assay, 5 μL of plasma from male pupfish was incubated for 19 h at 4°C with 11-KT AChE tracer and 11-KT ELISA antiserum in a 96-well plate pre-coated with mouse monoclonal anti-rabbit IgG. After incubation, the plate was developed with Ellman's reagent for 110 min and then read at 405 nm on a Victor X4 Microplate Reader (PerkinElmer, Waltham, MA, USA). The 11-KT ELISA standard curve spanned from 0.78 to 100 pg mL^−1^ 11-KT. The intra-assay coefficient of variability (% CV) was 4.8% for the E_2_ assay and 1.9% for the 11-KT assay.

### Gonadal histology

Fixed gonadal tissues were stored in 70% ethanol prior to paraffin wax embedding. Testes and ovaries were dehydrated and cleared in a graded series of ethanol followed by xylene, and then infiltrated with molten paraffin wax (Leica Biosystems TP 1020 tissue processor, Buffalo Grove, IL, USA). Embedded tissues were sectioned at 5 μm using a rotary microtome (Microm HM 325, Leica Biosystems, Buffalo Grove, IL, USA). Five serial sections were mounted on glass slides and stained using hematoxylin and eosin. Gonad sections were analyzed using bright-field microscopy (Olympus BX60, Olympus Americas, Center Valley, PA, USA), and micrographs were taken using a digital camera (QICAM QImaging Fast 1394, QImaging) and imaging software (Q-Capture Pro 7, QImaging, 2010).

Ovarian follicles were staged based on morphological characteristics previously established for fish ovarian development (Wallace and Selman 1981; Nagahama 1983; Guraya 1986; Wallace and Selman 1990). Ovarian follicle stages were classified using the following identifying criteria, beginning with the most premature stage: (1) perinucleolar stage was identified by dark purple hematoxylin staining and the absence of cortical alveoli; (2) cortical alveolus stage was characterized by the presence of a zone of cortical alveoli throughout the ooplasm; (3) lipid droplet stage with the presence of lipid droplets throughout the ooplasm identified as non-staining, clustered spherical structures; (4) tertiary/vitellogenic stage was enlarged follicles with vitellogenin and thick chorion; and (5) post-ovulatory follicles. The percentage of ovarian follicles at each stage of development was determined by classifying, counting, and calculating the percentage of all follicles in five tissue sections per female. Individual females were then also classified into one of three categorical reproductive states based on the most advanced developmental stage of follicle present in that female: “developing,” “mature,” or “spawning.” The state of reproduction was determined by the most advanced stage of ovarian follicles present. “Developing” individuals had ovaries with only perinucleolar and/or cortical alveolus stage follicles. “Mature” individuals had ovaries with perinucleolar, cortical alveolus, lipid droplet, and tertiary stage follicles. A “Spawning” female had all stages of ovarian follicle development as well as the presence of post-ovulatory follicles. Testes were staged for the proportion of spermatogonia, spermatocytes, spermatozoa, and empty vesicles. Based on which of those spermatogenesis stages were present in the testis, individual males were categorized as being in one of the following: “developing” with spermatocytes and early spermatid development only, “mature” with some (<50%) spermatids present, or “spawning” with tubules filled with spermatids, and all stages of spermatogenesis represented and/or evidence of previous spawning.

### RNA extraction and reverse transcription

Total RNA was extracted from pituitary, gonad, and liver tissue using TriReagent (Molecular Research Center, Cincinnati, OH, USA) using bromochloropropane as the phase separation reagent. The extracted RNA was DNase treated (Turbo DNA-*free*™ Kit, Thermo Fisher Scientific, Waltham, MA, USA) and quantified by spectrophotometry (Nano-Photometer® P300, Implen, Inc., Westlake Village, CA, USA). Total RNA was reverse transcribed in separate reactions for each tissue type that varied in RNA concentration and volume. RNA concentrations for reverse transcription were 10 ng/μL for pituitary, 10 ng/μL for gonad, and 57 ng/μL for liver. First-strand complementary DNA (cDNA) was synthesized by incubating total DNase-treated RNA template with GoScript™ reverse transcriptase (Promega Corp., Madison, WI, USA), 0.5 mM dNTPs (Promega Corp.), 0.5 μg/reaction oligo(dT) primers (Promega Corp.), 1 u/μL recombinant RNasin ribonuclease inhibitor (Promega Corp.), 2.5 mM MgCl2, 5× buffer (Promega Corp.), and nuclease-free H_2_O. All reverse transcription reactions were run under the following thermal profile: 25°C for 5 min and 42°C for 1 h, followed by 70°C for 15 min to inactivate the reverse transcriptase.

### Quantitative real-time PCR

Quantitative real-time PCR (qPCR) was performed following the guidelines outlined in Bustin et al. (2009). Gene-specific primers for real-time quantitative PCR reactions using SYBR^®^ Green intercalating dyes were either designed to cDNAs of *C. n. amargosae* that were amplified and sequenced using degenerate primer PCR as previously described (Lema et al. 2022), or designed to cDNA sequences identified from a cDNA library to *C. n. amargosae* generated by RNAseq (Novogene Corp., Sacramento, CA, USA). RNAseq was performed on combined liver, ovary, pituitary gland, and hypothalamus tissues from a sexually mature female Amargosa pupfish. RNAseq was conducted using mRNA purified from total RNA via poly-T oligo-attached magnetic beads, and first-strand cDNA was synthesized after fragmentation using random hexamer primers. Illumina NovaSeq 6000 (PE150) sequencing generated ∼57 million cDNA reads (GenBank BioProject number PRJNA866998; SRA number SRR20946675). Reads were assessed for quality using FastQC, and any remaining adapter sequences were removed from the reads using Cutadapt (Martin 2011) before assembling the reads into a de novo transcriptome using Trinity v. 2.9.1 (Grabherr et al. 2011). BLAST searches identified full-length cDNAs encoding *C. n. amargosae* follicle-stimulating hormone β-subunit (*fshβ*: 760 bp, GenBank accession number OP267480), luteinizing hormone β-subunit (*lhβ*: 627 pb, OP267479), steroid acute regulatory protein (*star*: 1829 bp, OP267484), and ovarian aromatase (*cyp19a1a*: 1895 bp, OQ283590), as well as partial cDNAs for the following genes: luteinizing hormone receptor (*lhcgr*: 363 bp, OP267482), follicle-stimulating hormone receptor (*fshr*: 3183 bp, OP267483), mitochondrial cholesterol side-chain cleavage enzyme P450scc (*cyp11a1*: 1740 bp, OP267485), 3β-hydroxysteroid dehydrogenase (*3βhsd*: 299 bp, OP267486), 11β-hydroxysteroid dehydrogenase (*11βhsd*: 193 bp, OP267487), 17β-hydroxysteroid dehydrogenase (*hsd17β3*: 272 bp, OP267489), and *shbg* (560 bp, OP267488). Partial transcripts encoding nuclear estrogen receptors *esr2a* (150 bp, OQ266774) and *esr2b* (1022 bp, OQ266775), as well as vitellogenin A (*vtga*) (1066 bp, OQ266776), and choriogenins L (*cgl*) (650 bp, OQ266777), H (*cgh*) (769 bp, OQ266778), and H-minor (*cghm*) (964 bp, OQ266779) were also identified within the transcriptome. For choriogenin genes *cgl* and *cghm*, SYBR assays were designed to the aligned sequences of the partial transcripts identified above using RNAseq, and partial cDNAs for those same genes *cgl* (KJ850333) and *cghm* (KJ850332) were isolated previously from *C. n. amargosae* using degenerate primer PCR and Sanger sequencing as described in Lema et al. (2022). Nucleotide sequences for those degenerate primer sets are provided in Johnson and Lema (2017). Primers for a SYBR™ Green qPCR assay for estrogen receptor *esr1* were designed to a consensus region in the gene identified by aligning a whole genome sequence (WGS) scaffold sequence (JSUU01001417) containing the *esr1* gene in the unannotated genome assembly of the closely related pupfish *C. n. pectoralis* (GenBank genome sequence number GCA_000776015) and *esr1* sequences from the pupfish, *C. variegatus* (XM_015400940 and XM_015369840) used previous to quantify *esr1* transcripts in Lema et al. (2022). Lastly, relative expression levels for several reference genes were also quantified, including elongation factor-1α (*ef1α*) (EU906930), 60S ribosomal protein L8 (*rpl8*) (KJ719257), β-actin (*actb*) (EU886377), and TATA-box binding protein (*tbp*) (OP222255). Partial cDNAs for those genes were either obtained from RNAseq as described above or amplified and sequenced using degenerate primers in prior studies (Lema 2010). Whenever possible, gene-specific primer sets for qPCR were designed to protein-coding regions of each gene transcript with primers designed to span an intron. Primers were synthesized by Eurofins MWG Operon (Huntsville, AL, USA), and nucleotide sequences for all primers are provided in Supplementary data, Table S1. Primer set specificity was confirmed by Sanger sequencing select PCR amplification products.

All qPCR reactions were conducted as 10 μL volumes containing 5 μL of PowerUp™ SYBR™ Green Master Mix (Thermo Fisher Scientific, Waltham, MA, USA), 0.65 μL each of forward and reverse primers (10 μM), 2.2 μL of RNase/DNase-free H_2_O, and 1.5 μL of reverse transcribed cDNA template. Reactions were run on a CFX96™ Connect Real-Time PCR System (BioRad Laboratories, Inc., Hercules, CA ) under a thermal profile of 95°C for 2 min and 42 cycles of 95°C for 10 s and 60°C for 30 s, followed by a melt curve analysis. Standard curves for qPCR were made for each tissue separately using RNA pooled from fish representing all treatment groups, and each standard was serially diluted and assayed in triplicate. DNA contamination was assessed by analyzing RNA samples that were not reverse-transcribed. Each qPCR run also included samples without cDNA as a further control. PCR efficiencies for each gene were calculated as % efficiency = [10(^1/slope^) − 1] ×100. Mean % efficiency values for each gene assayed are provided in Table 1. Due to variation in expression levels of reference genes by treatment for different tissues, tissue-specific combinations of reference genes were used for normalization to remove treatment differences in expression. For the pituitary gland, genes of interest were normalized by the geometric mean of *ef1a* and *rpl8*. Gonad genes were normalized by *tpb* only, and liver genes were normalized by the geometric mean of *actb* and *ef1a*. All relative mRNA abundance data were plotted as a relative level normalized to the mean value of that gene in same-sex pupfish from the constant, low temperature treatment conditions.

### Statistical analyses

Separate statistical analyses were performed for males and females. Temperature effects on GSI and HSI were analyzed for each sex using a one-way ANOVA. Female GSI data were square root transformed to meet parametric assumptions of ANOVA, and male GSI data were log transformed to correct non-normality and analyzed with Welch's ANOVA due to unequal variances of groups. Body condition values were calculated according to Fulton's body condition factor (*K*), where *K* = 100 × Mass (g)/Length (cm)^3^ (Fulton 1904). *K* values were analyzed using one-way ANOVA models for each sex. When significant (α < 0.05) effects were found for an ANOVA model, post-hoc Tukey HSD tests were then used to examine pairwise differences among treatment groups.

Plasma hormone values greater than two standard deviations (SDs) from the mean were removed as outliers resulting in the removal of two outliers from female E_2_ data and one outlier from male 11-KT data. Temperature effects on female plasma E_2_ levels were then analyzed using Welch's ANOVA model, as neither normality nor equal variance assumptions were met. Pairwise differences in female plasma E_2_ between treatment groups were assessed using the non-parametric Games–Howell post-hoc test. 11-KT levels in males were analyzed using a one-factor ANOVA.

Treatment differences in the proportions of gonadal reproductive states (developing, mature, and spawning) were assessed using contingency tables and a chi-square test for females and males. A repeated measures ANOVA was used to analyze how female ovaries varied in the proportions of five follicular stages across treatment groups. To test for treatment differences in the proportions of each follicular stage, one-factor ANOVAs were used with an α = 0.0033 per Bonferroni correction. A repeated measures ANOVA was also used to detect variation in proportions of four distinct stages of sperm development in the testes. Treatment differences of each spermatogenic stage were tested using one-factor ANOVAs and a Bonferroni corrected α = 0.0042.

For all gene expression data, group outliers greater than two SDs away from the mean were removed prior to performing statistical tests. In females, pituitary *fshb* expression data were square root transformed to meet test assumptions prior to ANOVA, while *lhb* was assessed using Welch's ANOVA due to unequal variances. Male *fshb* data did not meet the normality assumption of ANOVA, so were analyzed using the Kruskal–Wallis chi-square statistic, while *lhb* data were cube root transformed prior to ANOVA followed by post-hoc Tukey HSD pairwise comparisons. In females, *fshr, lhcgr, star, 11bhsd, cyp11a1, cyp19a1a*, and *shbg* in the ovary did not meet the normality assumption of ANOVA, so the non-parametric Kruskal–Wallis test was used, followed by the Wilcoxon rank sum test to analyze pairwise differences. Ovarian expression data for *3bhsd* and *hsd17b3* met the normality assumption but not equal variances, so Welch's ANOVA was used in place of ANOVA, and post-hoc treatment differences were determined using the Games–Howell test. Due to non-normality and low sample sizes in the *High* group, all testicular gene expression data were analyzed using the non-parametric Kruskal–Wallis.

The abundance of hepatic gene transcripts encoding *esr1, esr2a, esr2b, shbg, cgh, cghmin, cgl*, and *vtga* was examined in females only since previous studies on *C. n. amargosae* found that cDNA amplification of these transcripts using qRT-PCR was either undetectable or detected at exceedingly low levels in the livers of male fish (Lema et al. 2022). Due to their non-normal data distributions, all hepatic genes were assessed for treatment differences using the non-parametric Kruskall–Wallis test followed by the Wilcoxon rank sum test when pairwise analysis was applicable.

Data are presented as mean ± standard error of the mean (SEM) values. All statistical tests were two-tailed using an α = 0.05, unless described above otherwise. Statistical tests were performed using JMP Pro 15 software (SAS Institute, Inc.), except the Games–Howell post-hoc tests, which were calculated using IBM® SPSS® Statistics v. 29 software.

## Results

### Temperature effects on GSI, *K*, and plasma hormones

**Fig. 2:**
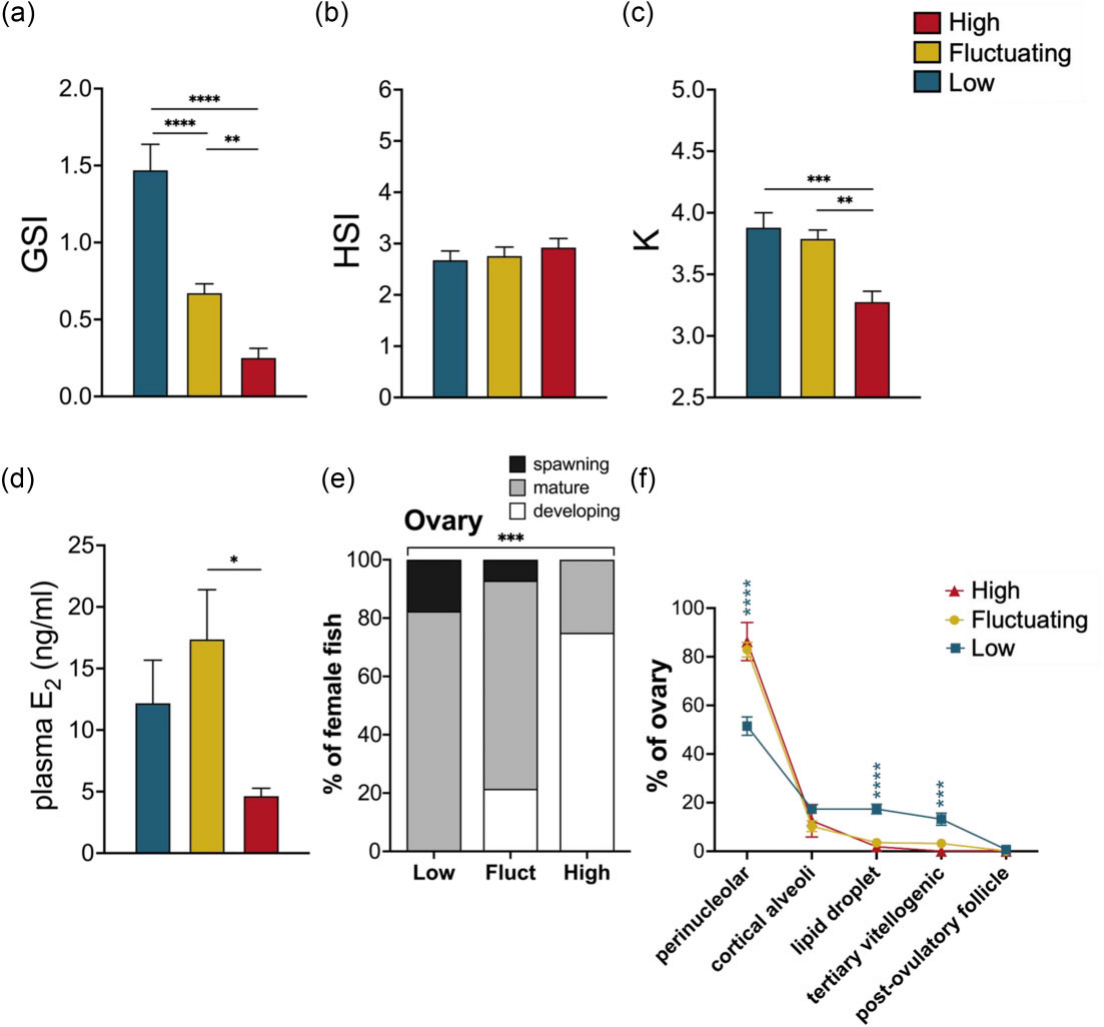
Effects of 50-day exposure to *Low* (25°C), *Fluctuating* (27–35°C), and *High* (35°C) temperature conditions on female gonadosomatic index (GSI) **(a)**, hepatosomatic index (HSI) **(b)**, Fulton's body condition factor (*K*) **(c)**, circulating 17β-estradiol (E_2_) levels **(d)**, proportions of ovarian reproductive states **(e)**, and ovarian percentages of five follicular gametogenic stages **(f)**. Data represent the mean ± SEM values (*n* = 8–19 fish/group). Asterisks indicate significant pairwise comparisons: *P ≤ 0.05, **P ≤ 0.01, ***P ≤ 0.001, and ****P ≤ 0.0001.

Following the 50-day exposure period, female pupfish body size remained similar across treatment groups (SL, *F*_2,39_ = 0.14, *P* = 0.87, body mass, *F*_2,39_ = 1.16, *P* = 0.32), while GSI exhibited significant reductions across treatments with increased high temperature exposure (Fig. 2a; *F*_2,38_ = 35.59, *P* < 0.001). Compared to those in *Low, Fluctuating* females had significantly smaller GSI values (*P* < 0.001) and those in *High* had the smallest ovaries relative to body size, significantly reduced compared to *Fluctuating* females (*P* = 0.001). Female HSI did not vary by treatment (Fig. 2b), and female body condition factor, *K*, exhibited temperature treatment differences (Fig. 2c; *F*_2,39_ = 8.34, *P* = 0.001) with reduced *K* in *High* females compared to both *Fluctuating* and *Low* groups.

Plasma E_2_ levels varied in female pupfish by treatment (Fig. 2d; *F*_2,17.33_ = 6.64, *P* = 0.0072) with significantly reduced levels in *High* females (4.63 ± 0.65 ng mL^−1^, mean ± SEM) compared to *Fluctuating*, which had the highest average concentration (17.36 ± 3.47 ng mL^−1^), while females in *Low* had intermediate E_2_ levels (12.17 ± 3.10 ng mL^−1^).

Male pupfish appeared to show few physiological effects from the different temperature regimes, and nearly all of the morphological and endocrine parameters quantified were found to be similar in males across the *Low, Fluctuating*, and *High* treatment conditions. GSI values did not vary among males from the different temperature conditions (Supplementary data, Fig. S1a), and plasma concentrations of 11-KT in male pupfish were similar across the treatments (Supplementary data, Fig. S1d). While *lhb* mRNAs were observed to be significantly higher in the pituitary gland of males from the *High* temperature condition (Supplementary data, Fig. S2b; *X^2^*_(2)_ = 7.42, *P* = 0.025), none of the other mRNAs quantified in the gland or in the testis varied among males from the three temperature treatments. Given those findings—and that male sample sizes for some of the analyses were reduced (see description in the *Materials and methods* section)—results for male pupfish are presented as online Supplementary data only.

### Temperature-associated variation in ovarian follicular stage

The ovaries of female pupfish showed significant variation in follicular staging across temperature treatments (Fig. 2e; *X^2^*_(4)_ = 19.61, *P* < 0.001). Compared to those in *Low*, females in *Fluctuating* were less frequently classified as being in a “spawning” state and instead had a higher proportion of “developing” ovaries. Very few of the female pupfish in the *High* condition had ovaries staged as “mature,” and none of the females in that *High* temperature regime were classified as “spawning,” as they showed few lipid droplet-staged follicles, and no evidence of tertiary vitellogenic or post-ovulatory follicles.

Ovaries exhibited significant variation in follicular developmental stages across temperature treatments (Fig. 2f; Wilks’ Lambda; Stage × Treatment effect, *F*_8,64 _= 6.26, *P* < 0.001). *Low* temperature females exhibited significantly lower proportions of perinucleolar stage follicles within their ovaries compared to females in the *Fluctuating* or *High* conditions (*F*_2,36_ = 20.63, *P* < 0.001). Conversely, the more advanced lipid droplet and tertiary vitellogenic follicular stages were present at significantly higher proportions in the ovaries of *Low* females than in the ovaries of *Fluctuating* or *High* females (lipid droplet: *F*_2,36_ = 27.71, *P* < 0.001; tertiary vitellogenic: *F*_2,36_ = 10.57, *P* < 0.001). The percentages of cortical alveoli and post-ovulatory follicular stages in ovaries did not differ across temperature treatments.

### Temperature effects on gene expression in the female pituitary, ovary, and liver

**Fig. 3:**
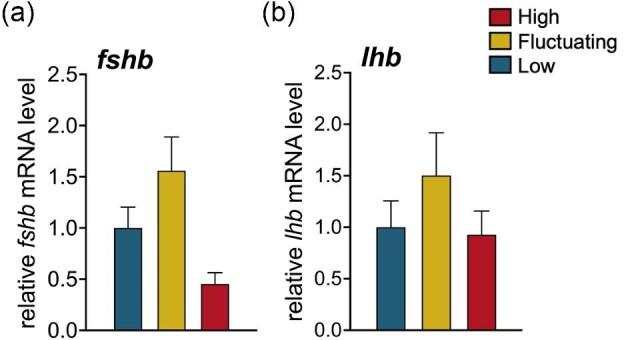
Relative expression levels of gene transcripts encoding pituitary gonadotropin hormones, FSHβ and LHβ, did not vary in females across *Low* (25°C), *Fluctuating* (27–35°C), and *High* (35°C) temperature conditions. Data represent the mean ± SEM values (*n* = 9–15 fish/group).

Variation in the relative abundances of pituitary gonadotropins, follicle-stimulating hormone (*fshb*), and luteinizing hormone (*lhb*), was not statistically significant across temperature treatments *Fluctuating* (Fig. 3a; *fshb*: *F*_2,34 _= 3.05, *P* = 0.061; Fig. 3b; *lhb*: *F*_2,22.94_ = 0.74, *P* = 0.45).

**Fig. 4:**
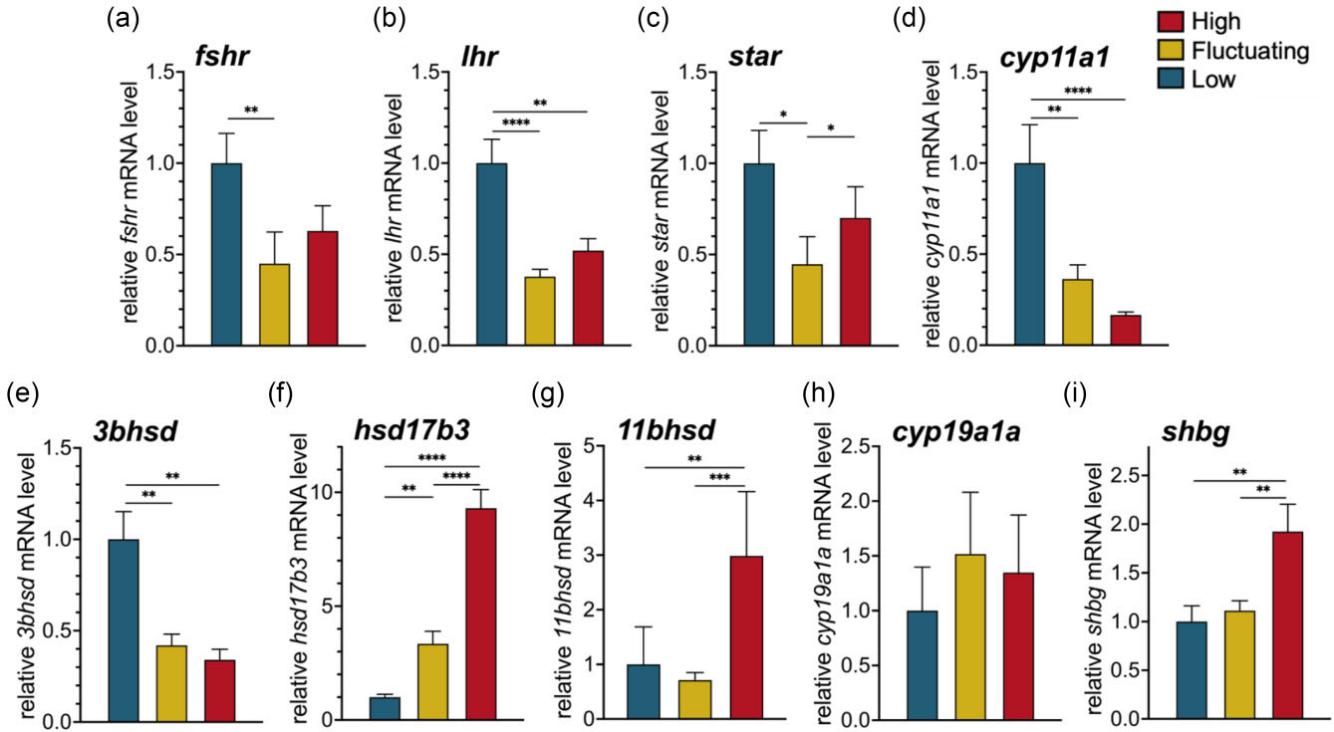
Temperature treatment effects on ovarian expression of gonadotropin receptor genes, follicle-stimulating hormone receptor (*fshr*) and luteinizing hormone receptor (*lhr*) (**a–b**), genes encoding six steroidogenic enzymes: steroid acute regulatory protein (*star*), P450 side chain cleavage (*cyp11a1*), 3-beta-hydroxysteroid dehydrogenase (*3bhsd*), 17-beta hydroxysteroid dehydrogenase 3 (*hsd17b3*), 11 beta-hydroxysteroid dehydrogenase (*11bhsd*), and ovarian P450 aromatase (*cyp19a1a*) (**c–h**), as well as the gene encoding the transport protein, sex hormone-binding globulin (*shbg*) (**i**). Data represent the mean ± SEM values (*n* = 8–16 fish/group). Asterisks indicate significant pairwise comparisons: *P ≤ 0.05, **P ≤ 0.01, ***P ≤ 0.001, and ****P ≤ 0.0001.

Temperature affected the relative expression levels of both follicle-stimulating hormone receptor (*fshr*) (Fig. 4a; *X^2^*_(2)_ = 10.36, *P* = 0.006) and luteinizing hormone receptor (*lhcgr*) (Fig. 4b; *X^2^*_(2)_ = 21.24, *P* < 0.001) in female ovaries. Gene transcripts of *fshr* and *lhcgr* were most abundant in the ovaries of females at *Low* temperature, and lowest in those from *Fluctuating* conditions (*fshr*: *P* = 0.001; *lhcgr*: *P* < 0.001). Compared to *Low* females, expression levels of *fshr* and *lhcgr* were also reduced in the *High* treatment, although this difference was only significant for *lhcgr* (*P* = 0.002).

The relative expression levels of several genes encoding proteins involved in ovarian steroidogenesis exhibited temperature-related variation. Gene transcripts encoding steroidogenic acute regulatory protein (*star*) varied with temperature (Fig. 4c; *X^2^*_(2)_ = 7.75, *P* = 0.021) with significantly lower amounts in females in the *Fluctuating* temperature group than either *Low* or *High*. Ovarian transcript levels of *cyp11a1* exhibited sensitivity to elevated temperatures (Fig. 4d; *X^2^*_(2)_ = 19.54, *P* < 0.001), with significant reductions in both *Fluctuating* and *High* treatment females compared to those under the *Low* condition.

Relative transcript abundances for *hsd3b, hsd17b3*, and *hsd11b* varied by temperature in distinct ways. Female mRNA levels of *hsd3b*, which encodes the enzyme that converts dehydroepiandrosterone (DHEA) to androstenedione, exhibited significant reductions under high temperature conditions (Fig. 4e; *F*_2,22.54_ = 8.02, *P* = 0.0023). Compared to females at *Low* temperature, *hsd3b* transcript abundances were lower in the ovary of both *Fluctuating* and *High* treatment females. In contrast, relative mRNA expression of *hsd17b3*, necessary for the conversion of androstenedione to testosterone, increased with elevated temperatures (Fig. 4f; *F*_2,14.14_ = 54.99, *P* < 0.001). *hsd17b3* was most elevated in females at *High* temperature—significantly more so than in *Fluctuating* or *Low* groups. Although reduced compared to *High*, levels of *hsd17b3* in the ovaries of females under the *Fluctuating* conditions were significantly higher than the *Low* group. Relative mRNA levels of *11bhsd* were highest in the ovary of females at *High* temperature (Fig. 4g; *X^2^*_(2)_ = 15.11, *P* < 0.001). Females exposed to the *Fluctuating* conditions had the lowest levels of *11bhsd*, resulting in the greatest difference occurring between the *High* and *Fluctuating* groups. *High* and *Low* groups also differed, but, unlike *hsd17b3*, there was no significant difference in *11bhsd* mRNA levels between females from the *Low* and *Fluctuating* temperature treatments. Notably, the gene encoding ovarian aromatase, *cyp19a1a*, which converts testosterone to E_2_, did not exhibit temperature-associated variation (Fig. 4h; *X^2^*_(2)_ = 0.84, *P* = 0.66).

Relative mRNA levels for *shbg* in the ovary varied with temperature treatment (Fig. 4i; *X^2^*_(2)_ = 10.63, *P* = 0.005). Females from the *High* temperature condition showed higher *shbg* mRNA levels in the ovary compared to females from either the *Fluctuating* or *Low* temperature treatments.

### Temperature effects on female liver gene expression

**Fig. 5:**
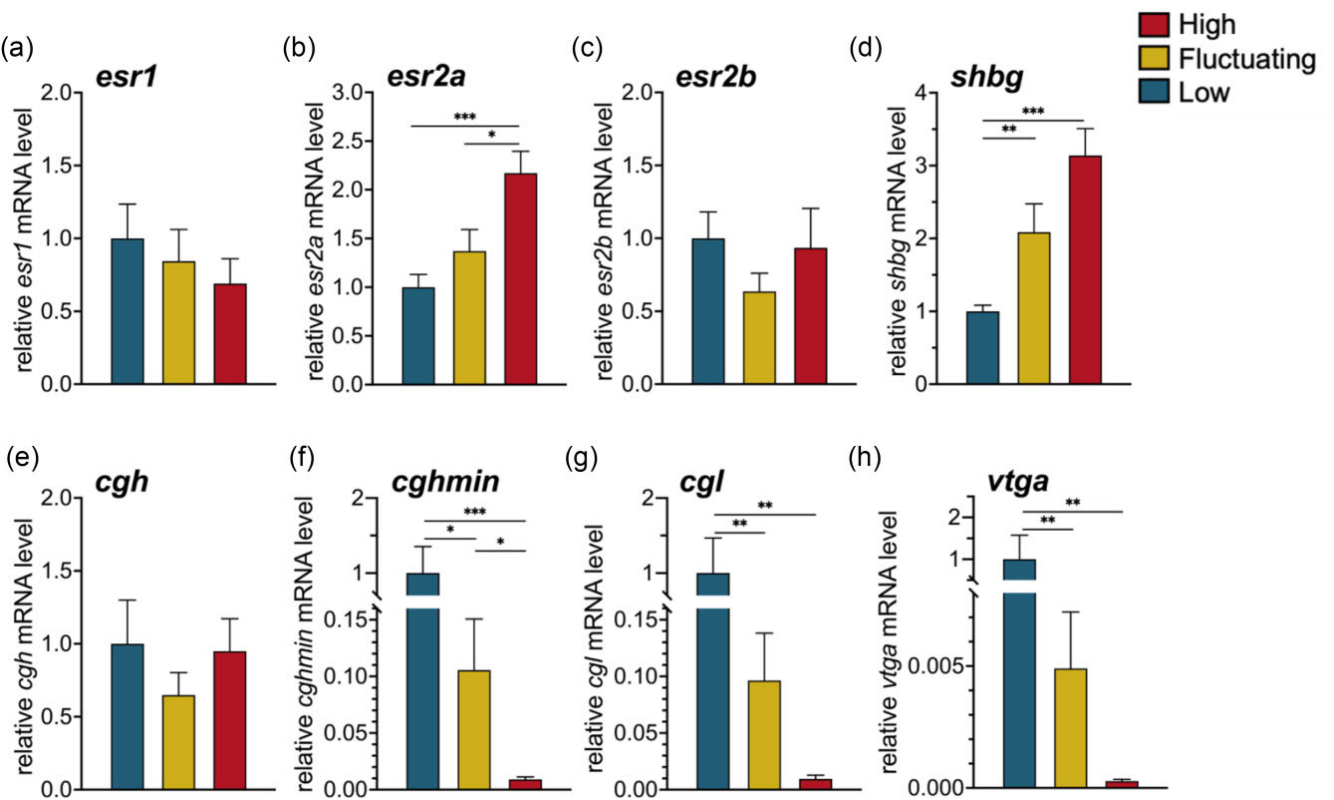
Temperature effects on liver gene expression in female pupfish held at *Low* (25°C), *Fluctuating* (27–35°C), and *High* (35°C) temperature conditions for 50 day. Relative mRNA levels were measured for genes encoding estrogen receptors (**a–c**), the transport protein, sex hormone-binding globulin (**d**), choriogenin precursor proteins (**e–g**), and the vitellogenin precursor protein (**h**). Data represent the mean ± SEM values (*n* = 9–14 fish/group). Asterisks indicate significant pairwise comparisons: *P ≤ 0.05, **P ≤ 0.01, and ***P ≤ 0.001.

Liver tissue from female pupfish was analyzed for the relative mRNA expression levels of eight genes involved in estrogen signaling or oogenesis. While transcript abundances of estrogen receptors 1 (*esr1*) and 2b (*esr2b*) did not vary by temperature treatment (Fig. 5a; *esr1*: *X^2^*_(2)_ = 0.37, *P* = 0.83; Fig. 5c; *esr2b*: *X^2^*_(2)_ = 1.52, *P* = 0.25), estrogen receptor 2a (*esr2a*) mRNA was elevated in liver tissue of females at *High* temperature (Fig. 5b; *X^2^*_(2)_ = 10.60, *P* = 0.005) compared to those in both *Fluctuating* and *Low* groups. Like *esr2a*, hepatic mRNAs for *shbg* increased in females experiencing high temperatures (Fig. 5d; *X^2^*_(2)_ = 19.05, *P* < 0.001). Specifically, compared to those females at *Low* temperature, hepatic *shbg* transcript levels were significantly elevated in both the *Fluctuating* and *High* temperature groups.

Expression levels of three genes encoding choriogenin egg envelope proteins also showed type-specific changes with temperature. Liver transcript abundances for choriogenin h (*cgh*) did not vary by temperature (Fig. 5e; *X^2^*_(2)_ = 1.21, *P* = 0.57), but mRNAs for *cghmin* (Fig. 5f; *X^2^*_(2)_ = 14.78, *P* < 0.001) and *cgl* (Fig. 5g; *X^2^*_(2)_ = 12.91, *P* = 0.002) were each significantly diminished in the livers of females experiencing the *Fluctuating* and *High* temperature conditions compared to females from the *Low* treatment. Hepatic mRNA abundances of *cghmin* and *cgl* were more reduced in females at *High* temperature than *Fluctuating*, but this difference was only significant for *cghmin* (*P* = 0.011). Relative transcript abundances associated with the egg yolk precursor protein *vtga* also exhibited reductions with high temperatures (Fig. 5h; *X^2^*_(2)_ = 13.17, *P* = 0.001). Hepatic *vtga* expression was measured to be highest in females held under the *Low* temperature condition with comparatively lower levels in those in *Fluctuating* and *High* temperature treatments.

## Discussion

Even eurythermal fishes with high thermal tolerances experience reproductive inhibition when exposed to elevated temperatures significantly lower than their thermal maxima (Shrode and Gerking 1977; Bock et al. 2021). Such reduced reproductive performance stems from changes in the production and action of hormones throughout the HPG axis that disrupt sex steroid production and gametogenesis (Bock et al. 2021; Lema et al. 2022). To date, however, most studies examining high temperature effects on the reproductive endocrinology of fishes have used stable or chronic temperature treatments; and whether the endocrine changes that occur from exposure to such constantly elevated temperatures are informative for predicting effects on fish experiencing intermittent exposure to high temperatures—as may be more likely in natural habitats—is not clear. Here, the effects of a 50-day exposure to either *High* (35°C), *Low* (25°C), or *Fluctuating* (varying 27–35°C diurnally) temperature conditions on sexually mature Amargosa River pupfish were examined with the aim of understanding the how frequent but temporary exposure to temperatures known to inhibit reproduction in this species potentially alters hormone production and signaling along the HPG axis.

**Fig. 6:**
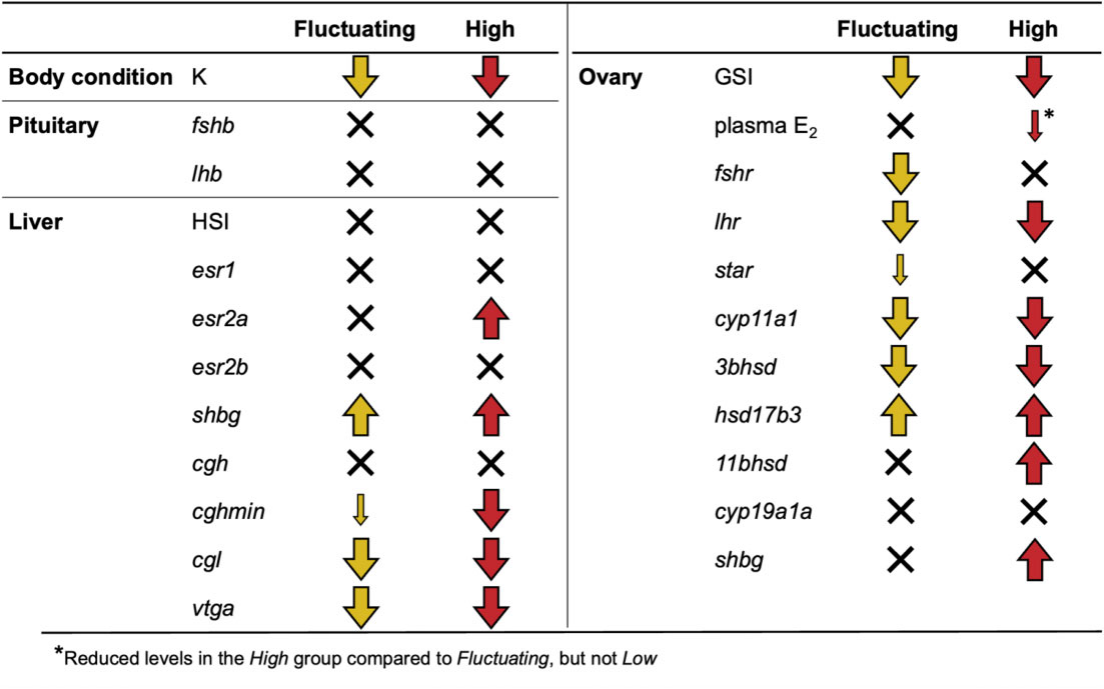
Summary of the effects of *Fluctuating* and *High* temperatures on female body condition (*K*), gonadosomatic and hepatosomatic indices (GSI and HSI), and gene expression in pituitary, liver, and ovarian tissues. Elevated temperatures associated with *Fluctuating* and *High* conditions did not affect pituitary mRNA levels, and responses in the liver and ovary were variable. Notably, transcript abundances of three out of four hepatic genes associated with oogenesis (*cghmin, cgl*, and *vtga*) exhibited significant reductions in the *Fluctuating* and *High* groups. Ovarian genes (right panel) are ordered in accordance with the gonadal steroidogenesis pathway, illustrating how genes involved earlier in this pathway largely exhibited reduced expression associated with both *Fluctuating* and *High* temperature exposure, while transcript levels of the latter steroidogenic genes either increased or did not change under these conditions. Arrow direction (up or down) indicates relative increases and decreases in *Fluctuating* and *High* groups compared to *Low*. Small arrows signify pairwise differences of 0.01 < *P* ≤ 0.05, large arrows indicate *P* ≤ 0.01, and black Xs indicate no significant differences.

Like what has previously been seen in *Cyprinodon* pupfishes (Bock et al. 2021; Lema et al. 2022), the reproductive physiology of female *C. n. amargosae* exhibited broader and more marked temperature effects than that of males. What is more, females in the *High* as well as *Fluctuating* temperature conditions experienced significant ovarian recession, had fewer spawning-capable ovarian follicles, and substantial reductions in relative levels of ovarian and liver gene transcripts encoding essential proteins in gonadal steroidogenesis and vitellogenesis. These females who underwent chronic or intermittent exposure to 35°C exhibited significant reductions in the relative abundances of ovarian gonadotropin receptor gene transcripts, *fshr* and *lhr*, steroidogenic enzymes, *cyp11a1* and *hsd3b*, as well as hepatic choriogenin and vitellogenin mRNAs, *cghmin, cgl*, and *vtga* (Summarized in Fig. 6). The downregulation of these genes combined with the significantly diminished ovaries in *Fluctuating* and *High* female pupfish suggests that fluctuating thermal regimes with brief, yet regular exposure to high temperatures can potentially induce reproductive disruption in females similar to the effects seen under chronic high temperature.

The effects of temperature were not consistent throughout the HPG axis components examined here. Female pituitary expression of both gonadotropin hormone genes, *fshr* and *lhr*, did not vary significantly across treatments. Ovarian mRNAs for HSD enzymes, *hsd17b3* and *11bhsd*, involved in the latter steps of steroidogenesis, exhibited significant upregulation under high temperature, but ovarian aromatase expression did not vary by treatment. Notably, *shbg* expression was significantly upregulated by high temperatures in both female ovaries and livers, with ovarian *shbg* greatest in *High* temperature females, and hepatic *shbg* significantly higher in both *Fluctuating* and *High* females compared to *Low*. Hepatic genes that did not exhibit significant temperature-related variation included estrogen receptors, *esr1* and *esr2b*, and choriogenin H, *cgh*. In contrast, male pupfish did not show any strong effects of temperature on testicular GSI or relative mRNA transcript abundances in the testis, implying that male reproduction was comparatively unaffected by the different temperature treatments. That finding agrees with what was observed previously with males of this species (Lema et al. 2022) and the related sheepshead minnow *C. variegatus* (Bock et al. 2021) where temperature effects on HPG axis endocrine signaling were also considerably more pronounced in females than in males.

### Temperature effects on female GSI, HSI, *K*, and ovarian follicles

The effects of temperature on GSI seen here demonstrate the susceptibility of female *C. n. amargosae* pupfish to experience significant ovarian regression under both chronic and intermittent high temperature scenarios. Compared to females held at *Low* temperature, the average GSI of females in the *Fluctuating* treatment was reduced by over half, and the ovaries of females held at *High* temperature were even smaller, averaging ∼⅙ that of the GSI recorded in *Low* females. Such diminished gonadal mass is consistent with a downregulation of ovarian steroidogenesis and oogenesis (Gillet 1991; Pankhurst et al. 1996; King et al. 2003; Gillet et al. 2011; Anderson et al. 2012a; Elisio et al. 2012), often accompanied by reduced fertility and higher proportion of atretic follicles (Dorts et al. 2012; Alix et al. 2020). Our results corroborate similar reductions in GSI as a result of prolonged exposure to elevated temperatures, as observed in three-spined stickleback (*Gasterosteus aculeatus* L.; Hani et al. 2019), European bullhead (*Cottus gobio* L.; Dorts et al. 2012), red seabream (*P. major*; Okuzawa and Gen 2013), and coho salmon (*O. kisutch*; Little et al. 2020). There are even cases of jeopardized ovarian function without relative declines in ovarian mass, as seen in pejerrey (*O. bonariensis*), which exhibited more atretic oocytes under higher temperature, absent a significant decline in ovarian GSI (Soria et al. 2008; Elisio et al. 2012). The closely related species to the Amargosa River pupfish, the sheepshead minnow (*C. variegatus*), exhibited reductions in ovarian GSI under elevated temperature conditions (37°C) accompanied by fewer spawning capable oocytes and more atretic oocytes in females (Bock et al. 2021).

### Temperature-related variation in female E_2_ production and its association with ovarian aromatase

Another gonadal effect often seen accompanying reduced GSI is deceased concentrations of circulating sex steroid hormones—the final products of gonadal steroidogenesis. Ovarian production of 17β-estradiol (E_2_) and its precursor hormone, testosterone (T), is crucial for oogenesis by stimulating hepatic production of oocyte chorion and yolk precursor proteins (Lubzens et al. 2010). In the current study on *C. n. amargosae*, females in the *Fluctuating* condition exhibited the highest plasma E_2_ levels, which varied significantly in comparison to the lowest average E_2_ levels of *High* females. While E_2_ was detected at a higher concentration in *Low* females than in *High*, this difference was not significant. More marked reductions in female E_2_ and T production under high temperature are nearly ubiquitous in 13 other species examined, apart from the lined seahorse, *Hippocampus erectus* (reviewed by Alix et al. 2020). Due to low blood plasma volumes recovered from individual pupfish, it was not possible to measure female circulating T levels in addition to E_2_.

The lack of variation in circulating E_2_ between females exposed to *Low* and *High* conditions used here may point to the ability for this species to maintain sufficient ovarian estrogen synthesis across that entire span of temperatures, or possibly to some degree of acclimation under longer duration exposure to consistent temperature conditions. The 35°C temperature is near the observed upper limit for spawning by *C. n. amargosae* in their Amargosa River habitat (Soltz 1974) and similar to the upper thermal limit of 36°C for egg production observed in captivity with the closely related subspecies *C. n. nevadensis* (Shrode and Gerking 1977). In that same subspecies of *C. nevadensis*, egg production, quality, and viability were all favorable across a temperature range of 24–30°C with spawning and hatching success most optimal at 28–30°C (Shrode and Gerking 1977). And, in a different study, the same parameters were tested on adult *C. n. nevadensis* acclimated to 24C, 28, and 32°C, with the 28°C group producing substantially more (i.e., higher fecundity) and higher quality eggs than females at the other temperatures (Gerking et al. 1979). It is thus possible that both the 25 and 35°C tested in this study were similarly suboptimal from the 28–30°C optimal range for egg production documented by Shrode and Gerking (1977), and thus resulted in no major impacts on E_2_ synthesis.

Supporting that idea, female pupfish in all three temperature treatments exhibited similar ovarian mRNA abundances for ovarian aromatase (*cyp19a1a*), despite the higher plasma E_2_ concentrations in females from the *Fluctuating* temperature conditions and clear differences in ovarian GSI values. Cyp19a1a converts androgens to estrogens (Simpson et al. 1994; Piferrer and Blázquez 2005; Young et al. 2005; Guiguen et al. 2010), and expression of this enzyme in the ovary has been found to be temperature sensitive in several fishes (e.g., Fernandino et al. 2008; Guiguen et al. 2010; Navarro-Martín et al. 2011). In a separate recent study with *C. n. amargosae*, females maintained in mixed-sex groups at either 24 or 34°C for 88 day likewise did not differ in ovarian *cyp19a1a* gene transcript abundance (Lema et al. 2022). Taken together, prior observation and our data here suggest that ovarian aromatase gene expression might not be altered by temperature differences in the range of ∼24–36°C in *C. n. amargosae* females—or at least not in *C. n. amargosae* females from their primary habitat: the Amargosa River.

Lema et al. (2022) also examined temperature effects on ovarian aromatase in female *C. n. amargosae* collected from another habitat, Tecopa Bore, and observed *cyp19a1a* mRNAs in the ovary of those females maintained for 88 day at 34°C to be similar to females originating from the Amargosa River (at both 24 and 34°C). However, ovarian aromatase mRNA levels in those Tecopa Bore females were greatly elevated in females maintained at 24°C. Tecopa Bore is a remarkably hot thermal spring for fish. The Tecopa Bore habitat is not natural and was created in 1967 when the Stauffer Chemical Company was drilled for mineral exploration and inadvertently hit an artesian aquifer. Water emerges from the ground at this site at ∼47.5°C within a spring pool, and then cools as it flows through a marsh (Naiman 1974; Lema et al. 2019). *Cyprinodon nevadensis amargosae* pupfish from the Amargosa River dispersed into Tecopa Bore sometime before 1970 and now differ from those in the Amargosa River in several metabolic and morphological attributes (Lema et al. 2016, 2019), at least some of which appear to be related to plasticity-first population divergence caused by the hot temperatures of Tecopa Bore. Elevated ovarian *cyp19a1a* abundance in Tecopa Bore *C. n. amargosae* at 24°C compared to 34°C may more reflect an upregulation of this enzyme at 24°C, rather than a downregulation at 34°C, given that *cyp19a1a* mRNA abundance at 34°C was similar to that of Amargosa River *C. n. amargosae* at both 24°C and 34°C (Lema et al. 2022). In any case, the variable responses of ovarian *cyp19a1a* transcript abundance to elevated temperature in recently isolated populations of *C. n. amargosae* pupfish suggest plasticity or local adaptation of E_2_ synthesis. More research is needed to resolve the endocrine mechanisms underlying those divergent responses of *cyp19a1a*’s varied responses, as well as the implications of those different responses for reproductive function.

In light of the lack of any temperature-related changes in ovarian *cyp19a1a* mRNA abundance in female *C. n. amargosae* in this study, the lower E_2_ seen in females at *High* temperature (35°C) was likely not caused by downregulated ovarian aromatase mRNA, as has been seen in other fishes (reviewed in Miranda et al. 2013; Alix et al. 2020). Even so, the lower GSI values of female pupfish in *Fluctuating* and *High* treatments still point to a downregulation of oogenesis under warmer conditions, and possible explanations for that change in GSI may be found in both a broader look at the HPG axis of those fish and the status of genes expressed in the liver that are crucial for oogenesis and regulated by E_2_.

### Effects of temperature on HPG axis and ovarian steroidogenesis pathways

The gonadotropin hormones Fsh and Lh stimulate gonadal synthesis of sex steroid hormones, including E_2_, which then drive oocyte development and maturation (Yaron et al. 2003; Lubzens et al. 2010). Here, pituitary mRNA transcript levels of *fshb* were found to be lower in females from the *High* treatment than from *Fluctuating*, which mirrored the effect of temperature on E_2_. Fsh facilitates the vitellogenic phase of oocyte development by inducing ovarian synthesis and secretion of E_2_, which in turn stimulates the production of vitellogenic proteins in the liver, that are then taken up from the blood by the oocytes (Wallace 1985; Nagahama 1994; Yaron et al. 2003). The fact that pupfish females in the *High* regime showed lower pituitary *fshb* mRNAs—but not those females from the *Fluctuating* regime (although this difference was not statistically significant)—points to chronic high temperature exposure inhibiting ovarian steroidogenesis in part by reducing pituitary production of Fsh.

A similar reduction in pituitary *fshb* expression was observed in female red seabream exposed to high temperature for 5 day, but this effect disappeared by 10 day of high temperature exposure (Okuzawa and Gen 2013). Conversely, pituitary *lhb* mRNA levels in female red seabream were significantly reduced after exposure to that same elevated temperature for 10 day (Okuzawa and Gen 2013). Here in pupfish, however, we observed no change in female *lhb* expression after 50 day of elevated temperature exposure. While Fsh functions in the earlier stages of gametogenesis, Lh is involved in final oocyte maturation and ovulation (Yaron et al. 2003). These hormones not only play different functional roles in the gametogenic phase of reproduction but also have different feedback responses to the production of sex steroids (Yaron et al. 2003; Okuzawa and Gen 2013).

In male fishes, pituitary gonadotropins stimulate the production of E_2_ and 11-KT in the testes, driving spermatogenesis (Yaron and Levavi-Sivan 2011). Male *fshb* expression was unaffected by temperature, but relative levels of *lhb* were significantly higher in males at *High* temperature than either *Fluctuating* or *Low*. This contrasts with effects seen in male sheepshead minnows, where 14 day of 37°C exposure resulted in significant reductions in pituitary *fshb* and *lhb* mRNA levels (Bock et al. 2021). Notably, however, the sheepshead minnow in that study had been bred in captivity at 25°C and raised at that temperature through adult development, at which time they were exposed to 37°C. Relative mRNA levels for gonadotropin inhibitory hormone (*gnih*) in the hypothalamus were also observed to be increased in both sexes of those sheepshead minnows at 37°C (Bock et al. 2021). Although hypothalamic genes were not examined here, the reduced expression of *fshb* in female Amargosa River pupfish at *High* temperature in this study may also have resulted in part from upregulated hypothalamic levels of *gnih*, an inhibitor of pituitary LH and FSH secretion (Zhang et al. 2010; Tsutsui et al. 2012; Paullada-Salmeron et al. 2016, 2017; Muñoz-Cueto et al. 2017; Aliaga-Guerrero et al. 2018; Tsutsui and Ubuka 2018), or other alterations to hypothalamic HPG axis regulation, such as suppression of GnRH production, as has been seen in other teleost fishes at higher temperatures (reviewed in Zahangir et al. 2022).

Temperature effects on gonadal steroidogenesis were examined by comparing the expression levels of genes encoding for gonadotropin receptors, Star, and several steroidogenic enzymes in both sexes of pupfish. While no differences in gonadotropin receptor or steroidogenic pathway gene expression were detected in the testis related to temperature conditions, widespread temperature effects on mRNA expression levels were observed in the ovary. Gonadal steroidogenesis is stimulated by gonadotropin hormones binding to their respective receptors, Lhcgr and Fshr (Kumar and Trant 2001). Compared to females at *Low* temperature, those in the *Fluctuating* treatment showed reduced transcript abundances for both gonadotropin receptor genes *fshr* and *lhcgr*, while females maintained at *High* temperature also showed lower *lhcgr* mRNA abundance in the ovary compared to those at *Low*. Reductions in ovarian gonadotropin receptor expression at high temperatures have been observed in other fishes, including pejerrey, sheepshead minnow, and coho salmon (Soria et al. 2008; Elisio et al. 2012; Anderson et al. 2019; Bock et al. 2021), as well as in two populations of Amargosa River pupfish from distinctly different thermal habitats (Lema et al. 2022). In a study assessing the effects of two daily fluctuating high temperature scenarios on pejerrey, females in both fluctuating treatments experienced significant reductions in transcript levels of *fshr* and *lhcgr* compared to those held at a lower control temperature (Elisio et al. 2012). Here, the significant reduction in gonadotropin receptor gene transcript levels in female pupfish in *Fluctuating* suggests that even episodic exposures to temperatures exceeding those in the reproductive range may impact gonadal steroidogenesis by altering gonadotropin stimulation of the ovaries (Planas and Swanson 2008; Levavi-Sivan et al. 2010).

Following gonadotropin stimulation of the gonad, cholesterol is mobilized from the outer to the inner mitochondrial membrane via Star and then is subsequently converted to pregnenolone by the Cyp11a1 enzyme (Stocco et al. 2005; Nunez and Evans 2007). This transport of cholesterol into the mitochondria for conversion to pregnenolone is a rate-limiting step (Simpson and Boyd 1966; Wang et al. 1998; Stocco 2001) and the first event of steroidogenesis (Tenugu et al. 2021). Here, we observed that gene transcript levels of *star* were significantly reduced in the ovary of pupfish from *Fluctuating* compared to both *Low* and *High*. Additionally, expression of *cyp11a1* was at lower relative levels in both *Fluctuating* and *High* groups compared to *Low*. These results indicate a downregulation of cholesterol conversion to pregnenolone, which may contribute to the reduced E_2_ observed in females who experienced *High* temperature. Reductions in *star* expression have also been previously noted in female Nile tilapia (*Oreochromis niloticus*) and sheepshead minnow experiencing high temperatures (Mahmoud et al. 2020; Bock et al. 2021). However, in Atlantic salmon (*Salmo salar*), high temperature exposure did not alter transcript abundance of *star* (Anderson et al. 2017), but did lead to lower ovarian *cyp11a1* mRNA levels in females at later stages of vitellogenesis (Anderson et al. 2012b).

Like temperature effects on *cyp11a1*, gene transcript levels of the steroidogenic enzyme *hsd3b*, which converts DHEA to androstenedione, were reduced in the ovary of female pupfish from *Fluctuating* and *High* treatments. Contrary to this reduced *hsd3b* expression, *hsd17b3*, which encodes the steroidogenic enzyme that converts androstenedione to testosterone (T), was most highly expressed in females from *High*, and was also significantly elevated in the *Fluctuating* female ovaries compared to those in *Low*. Relative expression levels of *hsd11b*, the enzyme that subsequently converts T to 11-KT (Kusakabe et al. 2003; Ozaki et al. 2006), were also substantially elevated in females at *High* temperature, with no significant difference between *Fluctuating* and *Low* temperature groups. Since T is also a precursor to E_2_ synthesis, any increase in protein expression or activity for Hsd11β in the ovary of females at elevated temperatures might contribute to a reduction in E_2_ production in those females. Alternatively, the upregulation of Hsd11β in ovarian granulosa or theca cells has been proposed as a mechanism to protect the oocyte from cortisol (Faught and Vijayan 2018), as Hsd11β also converts cortisol to the less active 11-keto forms of glucocorticoids cortisone and 11-dehydrocorticosterone. What is more, Hsd11β has been identified as playing a role in the masculinization of fish at elevated temperatures. Under conditions of high temperature, cortisol increases triggered by the warm conditions upregulate gonadal *hsd11b* expression, which enhances 11-KT production with the ultimate effect being masculinization of the differentiating gonad and fish's overall phenotype (e.g., Hayashi et al. 2010; Fernandino et al. 2012; Goikoetxea et al. 2017). Whether the elevated ovarian *hsd11b* mRNAs observed here in female pupfish held at High (35°C) are related to temperature-related changes or have any effects on circulating E_2_ remains to be determined but should be examined in future studies.

### *shbg* upregulation under elevated temperatures

Shbg is a plasma glycoprotein that binds androgens and estrogens, modulating the bound: free (unbound) ratio of sex steroids and thus, their bioavailability to target tissues while in circulation (Bobe et al. 2010; Marivin et al. 2014; Laurent et al. 2016). The primary form of Shbg in fishes, Shbga, is produced in the liver, but another form of this protein, Shbgb, is expressed in the gonads and has been characterized in salmonid species (Bobe et al. 2010). In accordance with its role in regulating the bioavailability of circulating sex steroids, Shbg expression in fishes is variable during gonadal sex differentiation in early development as well as periods of reproduction (Miguel-Queralt et al. 2007; Marivin et al. 2014). A study on spotted sea trout (*Cynoscion nebulosus*) measured continuous increases in plasma Shbg levels throughout vitellogenesis to reach peak values during final oocyte maturation. However, these changes in plasma Shbg were not directly correlated with plasma levels of either T or E_2_ (Laidley and Thomas 1997). Conversely, decreases in plasma Shbg during sexual maturation were detected in sea bass *Dicentrarchus labrax*, and as those changes in Shbg were coincident with fluctuations in body composition, the decreased Shbg levels were thought to be related to nutritional or metabolic effects in relation to water temperatures and food intake, rather than changes in gonadal sex steroid production (Miguel-Queralt et al. 2007).

As of yet, there has only been one study testing the effects of elevated temperature on Shbg expression in a eurythermal fish. When sheepshead minnows were exposed to either 27 or 37°C for 14 day, females from the 37°C condition were found to have lower ovarian *shbg* mRNA abundance, but a greater relative abundance of *shbg* transcripts in the liver, than females at 27°C (Bock et al. 2021). Here, *shbg* mRNAs were significantly elevated in both the gonads and livers of females held at *High* temperature (35°C) treatment. In the ovaries, there was no difference between *shbg* mRNA levels in the *Low* and *Fluctuating* treatments, and comparatively elevated expression in *High* temperature females. Hepatic *shbg* transcripts were significantly higher in the livers of both *Fluctuating* and *High* temperature treatment females compared to *Low*. Those differences in liver *shbg* transcript abundance could be indicative of the high temperature response in eurythermal fishes, but it is not clear whether such increased mRNA expression is advantageous to the bioavailability and/or transport of E_2_ or other sex steroids, despite Shbg being a key binding protein for those hormones. More studies incorporating measurements of circulating Shbg and sex steroids will be needed to elucidate the reproductive consequences of the hepatic and ovarian *shbg* mRNA variation observed here.

### Reduced expression of hepatic genes linked to oocyte development

The liver plays a prominent role regulating oogenesis in conjunction with the ovaries. Under stimulation by E_2_ from blood circulation, hepatocytes synthesize vitellogenin precursors to egg yolk proteins, as well as choriogenin egg envelope precursors (Bowman et al. 2000; Folmar et al. 2000; Denslow et al. 2001a, [Bibr bib16][Bibr bib16]), which are both released from the liver into blood circulation and then sequestered from circulation by developing oocytes (Lubzens et al. 2010; Yaron and Levavi-Sivan 2011; Hara et al. 2016). Numerous studies have documented reductions in liver production of vitellogenin and choriogenin proteins in fishes experiencing high temperature (Pankhurst and Munday 2011; Mahanty et al. 2019; Bock et al. 2021; for review, see Alix et al. 2020). Reduced liver synthesis of vitellogenins and choriogenins can arise from lower circulating E_2_, or from an altered capacity of the liver to respond to E_2_ due to changes in estrogen receptor expression (e.g., Pawlowski et al. 2000; Watts et al. 2005). As a result, oogenesis can be impaired via reduced egg numbers or the production of lower quality eggs with insufficient yolk or malformed envelopes, as has been seen in *C. nevadensis* pupfish exposed to temperatures greater than ∼32°C (Shrode and Gerking 1977; Gerking et al. 1979; Gerking and Lee 1983).

Two prior studies have examined liver estrogen receptor expression in *Cyprinodon* pupfishes exposed to environmental temperature variation. Of three genes encoding for estrogen receptors in female sheepshead minnow, only hepatic estrogen receptor α (*esr1*) gene expression was found to be reduced in fish at elevated temperature, and no differences were observed in liver mRNA abundances for estrogen receptor genes *esr2a* or *esr2b* (Bock et al. 2021). In *C. n. amargosae* from the Amargosa River, transcripts encoding *esr1, esr2a*, and *esr2b* were previously observed to be at lower reduced relative abundances in the liver of females maintained for 88 day at 34°C compared to 24°C (Lema et al. 2022). Contrasting with those previous findings in *C. n. amargosae*, here transcripts encoding *esr2a* was observed to be elevated in female pupfish experiencing the *High* temperature condition for 50 day, compared to both *Low* and *Fluctuating* temperature regimes, but no effects of temperature were detected for liver *esr1* or *esr2b* mRNA abundance. While it appears clear that temperature conditions can alter hepatic estrogen receptor expression in female fish, the patterns of those effects appear highly variable, possibly due to differences in the thermal regimes tested or taxonomic variation in the functional role(s) of the estrogen receptors. In female European eel (*Anguilla anguilla*), for instance, liver *esr1* mRNA levels varied in accordance with the stage of ovarian oogenesis and showed temperature-dependent downregulation, but only at the early vitellogenic stage of oogenesis (Pérez et al. 2011).

Perhaps more directly informative for understanding temperature effects on female fertility, we observed that transcripts encoding both vitellogenin A (*vtgA*) and the choriogenin genes *cghmin* and *cgL* in the liver showed reductions in relative abundances of 90% or more in female pupfish in both the *Fluctuating* and *High* temperature conditions compared to females at *Low*. Such temperature-mediated declines in liver vitellogenin and choriogenin mRNA abundance were also previously observed in *C. n. amargosae* and *C. variegatus* pupfishes (Bock et al. 2021; Lema et al. 2022), as well as in other fishes (e.g., King et al. 2003; Pankhurst and Munday 2011; Anderson et al. 2012b). Since vitellogenin and choriogenin protein production has a direct functional connection to oocyte development and quality, the strong downregulation of those genes seen in females experiencing chronic (*High*) and intermittent (*Fluctuating*) exposure to 35°C suggests that these fish would likely experience declines in egg quality or quantity relative to those in the unchanging 25°C (*Low*) condition. That pattern likely explains the findings of Shrode and Gerking (1977), who observed that the few eggs spawned by *C. n. nevadensis* pupfish at temperatures of 32°C and above were smaller, had underdeveloped chorions, and largely lacked yolk.

## Conclusion

Resolving the variability in responses of teleost reproductive endocrine systems to thermal challenges is crucial to determining how fish populations will respond to warming temperatures under climate change. Here, we observed that both chronic and intermittent exposure to temperatures exceeding the upper limit of reproduction had pervasive impacts on reproductive endocrine regulation in female Amargosa River pupfish (summarized in Fig. 6). Male pupfish, in contrast, were largely unaffected by temperature, suggesting that female pupfish are more vulnerable to reproductive impairment at higher temperatures than male pupfish.

Supporting that idea, female GSI was substantially reduced under both *Fluctuating* and *High* temperature conditions with adjoining alterations in the relative expression levels of genes at multiple levels of the HPG axis, associated with steroidogenesis and gametogenesis (Fig. 6). As one of the first studies to explore how fluctuating temperature regimes impact reproductive regulation, our results demonstrate that even sporadic exposure to elevated temperature has notable effects on endocrine regulation of reproduction in female pupfish that have the potential to jeopardize reproductive function. And yet, despite the extensive disruptions to the expression levels of genes involved in steroid hormone production experienced by females from both fluctuating and high temperature treatments, E_2_ levels were higher in the plasma of *Fluctuating* group females compared to *High*. An explanation for this disparity in E_2_ production is not obvious, as relative levels of the gene encoding ovarian aromatase (*cyp19a1a*) the enzyme that converts testosterone to E_2_ did not differ by temperature treatment—a contrasting outcome from strong *cyp19a1a* thermal sensitivity observed in adults of other species (*O. bonariensis*, Elisio et al. 2012; *S. salar*, Anderson et al. 2012b; *A. anguilla*, Mazzeo et al. 2014; *O. kisutch*, Anderson et al. 2019). Even so, this outcome is consistent with Lema et al.’s (2022) finding that relative *cyp19a1a* expression was also not temperature sensitive in pupfish from the Amargosa River. As a shallow desert stream, the Amargosa River experiences wide fluctuations in temperature between day and night (Lema et al. 2019), and it is possible that the lack of *cyp19a1a* mRNA changes with temperature observed here reflect an adaptive mechanism for reproduction under those extraordinarily dynamic thermal conditions. Notably, however, the relatively higher amount of circulating E_2_ in *Fluctuating* treatment females was not consistent with hepatic endocrine functionality. Exposure to both *High* and *Fluctuating* temperatures yielded substantially reduced liver expression of vitellogenin and choriogenin precursor protein transcripts, which are incorporated into the structure of developing oocytes. The declines in GSI and hepatic expression of egg protein precursor genes occurred in females at *High* temperature in conjunction with lower plasma E_2_, but also occurred in females from the *Fluctuating* treatment despite E_2_ levels that were no different than 25°C females. This suggests that the liver might be experiencing inhibited function independent of estrogen stimulation. More research will be needed to resolve the underlying cause of inhibited liver function with high temperature exposure. But even so, our findings here provide evidence that fluctuating thermal regimes that only temporarily reach high temperatures beyond those of some critical range have the potential to affect female pupfish reproduction.

## Supplementary Material

obae003_Supplemental_Files

## Data Availability

RNAseq sequencing data generated by this research are available on NCBI as GenBank BioProject number PRJNA866998, and individual cDNA sequences are available on NCBI via accession numbers provided within the manuscript. Additional data that support the findings of this study are available on request from the corresponding author.
