# Supplementary material for: Fluctuating and Stable High Temperatures Differentially Affect Reproductive Endocrinology of Female Pupfish
Source: Integr Org Biol. 2024 Feb 1;6(1):obae003. doi: 10.1093/iob/obae003 (PMC10924253; doi:10.1093/iob/obae003)
Supplement: obae003_Supplemental_Files [file obae003_supplemental_files.zip › Cna - Supp materials - main body.docx]

**Results for male pupfish by temperature treatment**

Average male body size remained similar across treatment groups following experimental conditioning (SL, F_2,45_ = 0.52, *p* = 0.60; body mass, F_2,45_ = 1.42, *p* = 0.25). Unlike in females, male GSI did not exhibit temperature dependent variation (**Fig. S1a**; *F*_2,6.74_ = 4.08, *p* = 0.069). Male HSI also remained the same across treatment groups (**Fig. S1b**; *F*_2,42_ = 1.77, *p* = 0.18) but there was a significant reduction in body condition factor, K (**Fig. S1c**; *F*_2,45_ = 3.48, *p* = 0.039) in *High* temperature males compared to those in *Low* temperature (*p* = 0.044). Concentrations of 11-KT measured in the plasma of male pupfish (**Fig. S1d**; *F*_2,44_ = 3.06, *p* = 0.057) did not differ by temperature treatment. Nor did the proportions of the testicular reproductive states, ‘developing’, ‘mature’, or ‘spawning’ in each treatment group, as determined by histological examination (**Fig. S1e**; *X^2^*_(2)_ = 0.21, *p* = 0.90). However, there was significant variation in the percentage of each spermatogenic stage by treatment (**Fig. S1f**; Stage × Treatment effect, Wilks’ Lambda, F_6,40_ = 3.43, *p* = 0.008), although separate examinations treatment variation at each spermatogenic stage did not reveal differences.

In males, temperature treatment did not affect relative expression of *fshb* (**Fig. S2a**; *X^2^*_(2)_ = 1.36, *p* = 0.51), but did alter pituitary expression of *lhb* mRNA abundance (**Fig S2b**; *X^2^*_(2)_ = 7.42, *p* = 0.025). Males from the *High* temperature condition had a significantly greater relative abundance of *lhb* mRNAs in the pituitary gland compared to those in *Low* (*p* = 0.0034) and *Fluctuating* (*p* = 0.010) treatments. In male pupfish, the relative mRNA abundance of gonadotropin receptors *fshr* and *lhcgr* was similar across temperature treatments (**Fig. S3a,b**). Similarly, gene transcript abundances for *star* and *cyp11a1* were also unaffected by the temperature treatments used in this experiment (**Fig. S3c,d**). The absence of temperature effects on testicular steroidogenic gene expression also held for the steroidogenic enzyme genes *3bhsd*, *hsd17b3,* *11bhsd*, and *cyp19a1a*, none of which showed any statistically significant changes in relative mRNA levels in males (**Fig. S3e-h**). In accordance, relative mRNA levels for *shbg* in the testis were also unaffected by temperature conditions (**Fig S3i**).
