# Supplementary material for: Fluctuating and Stable High Temperatures Differentially Affect Reproductive Endocrinology of Female Pupfish
Source: Integr Org Biol. 2024 Feb 1;6(1):obae003. doi: 10.1093/iob/obae003 (PMC10924253; doi:10.1093/iob/obae003)
Supplement: obae003_Supplemental_Files [file obae003_supplemental_files.zip › Cna pupfish manuscript - supplementary figures.docx]

**
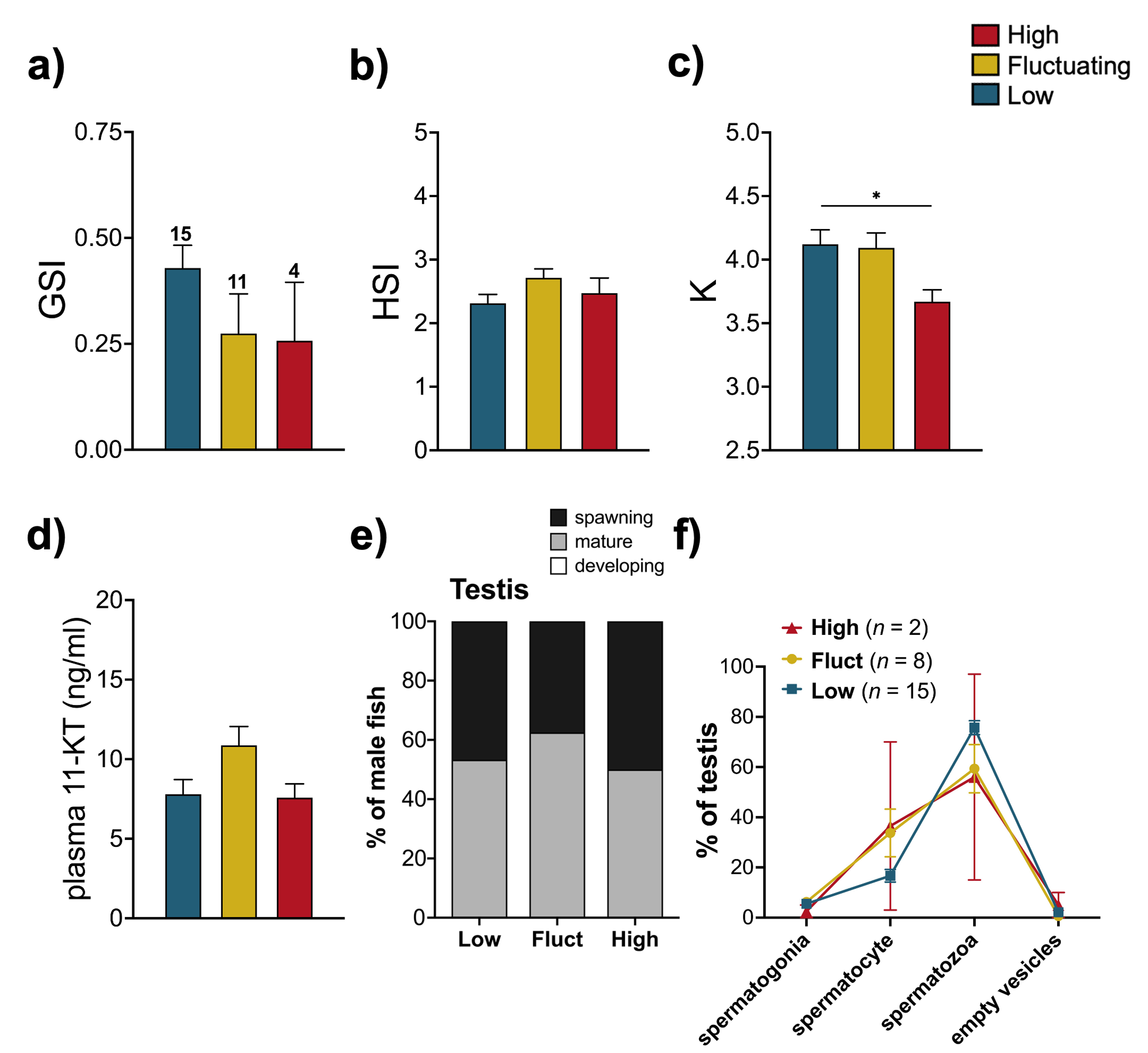
**

**Fig. S1.** Effects of 50 d exposure to *Low* (25°C), *Fluctuating* (27-35°C), and *High* (35°C) temperature conditions on male gonadosomatic index (GSI) **(a)**, hepatosomatic index (HSI) **(b)**, Fulton’s body condition factor, K **(c)**, circulating 11-ketotestosterone (11-KT) levels **(d)**, proportions of testicular reproductive states **(e)**, and testicular percentages of four spermatogenic developmental stages **(f)**. Data represent the mean ± SEM values (*n* = 2-19 fish/group and are indicated on figures were group *n*-values are <5). Asterisks indicate significant pairwise comparisons: * p ≤ 0.05, ** p ≤ 0.01, *** p ≤ 0.001, and **** p ≤ 0.0001.

**
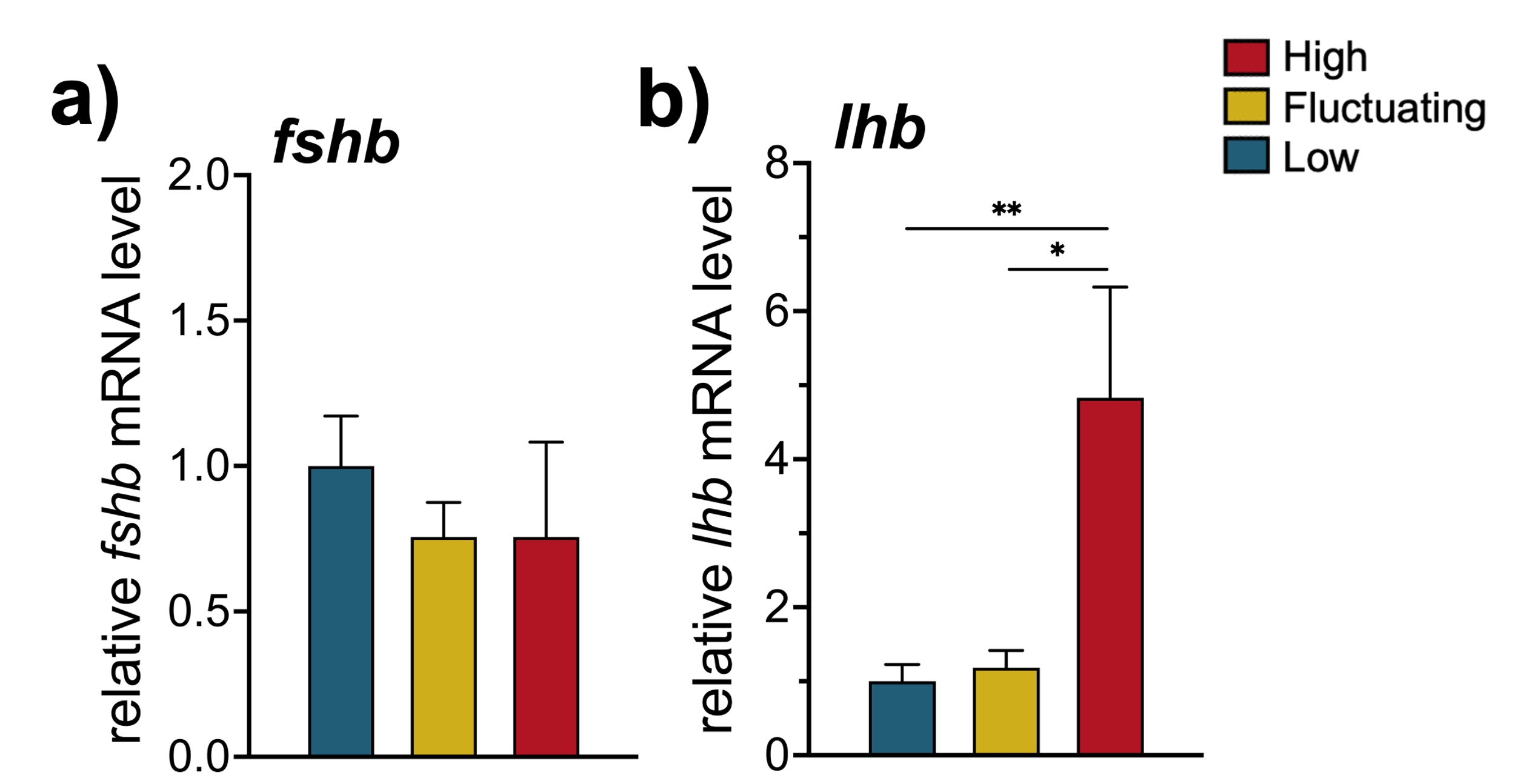
**

**Fig. S2.** Relative expression levels of gene transcripts encoding pituitary gonadotropin hormones, FSHβ and LHβ in male pupfish exposed to *Low* (25°C), *Fluctuating* (27-35°C), and *High* (35°C) temperature conditions. Data represent the mean ± SEM values (*n* = 8-17 fish/group). Asterisks indicate significant pairwise comparisons: * p ≤ 0.05, ** p ≤ 0.01

**
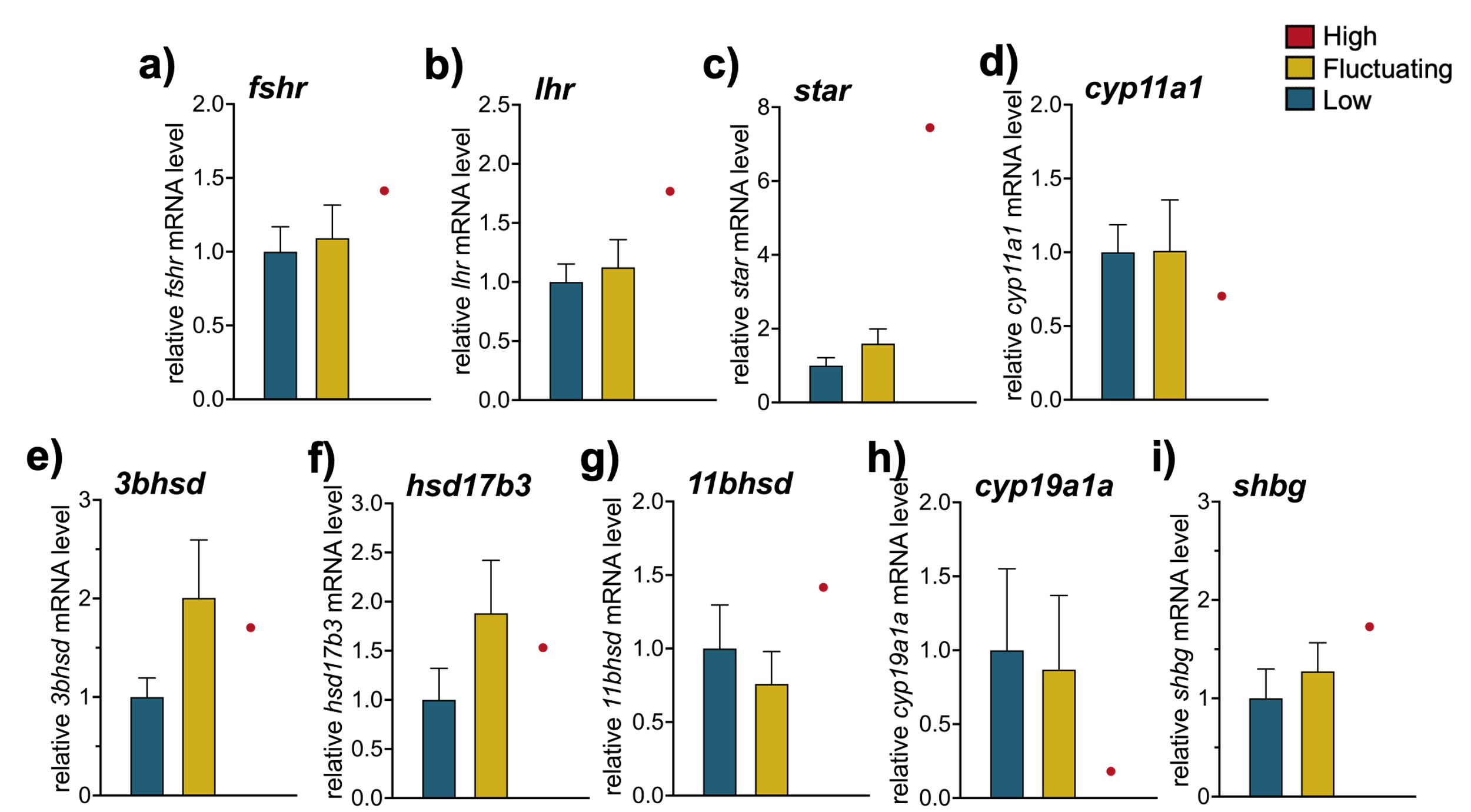
**

**Fig. S3.** In males, temperature did not significantly affect the expression of any of the nine gonadal genes measured in the testes. Data are shown as mean ± SEM values. *n* = 7-15 fish/group for *Low* and *Fluctuating* groups and *n* = 1 for *High*.
