# Supplementary material for: Fluctuating and Stable High Temperatures Differentially Affect Reproductive Endocrinology of Female Pupfish
Source: Integr Org Biol. 2024 Feb 1;6(1):obae003. doi: 10.1093/iob/obae003 (PMC10924253; doi:10.1093/iob/obae003)
Supplement: obae003_Supplemental_Files [file obae003_supplemental_files.zip › Cna pupfish manuscript - Table S1.pptx]

## Slide 1
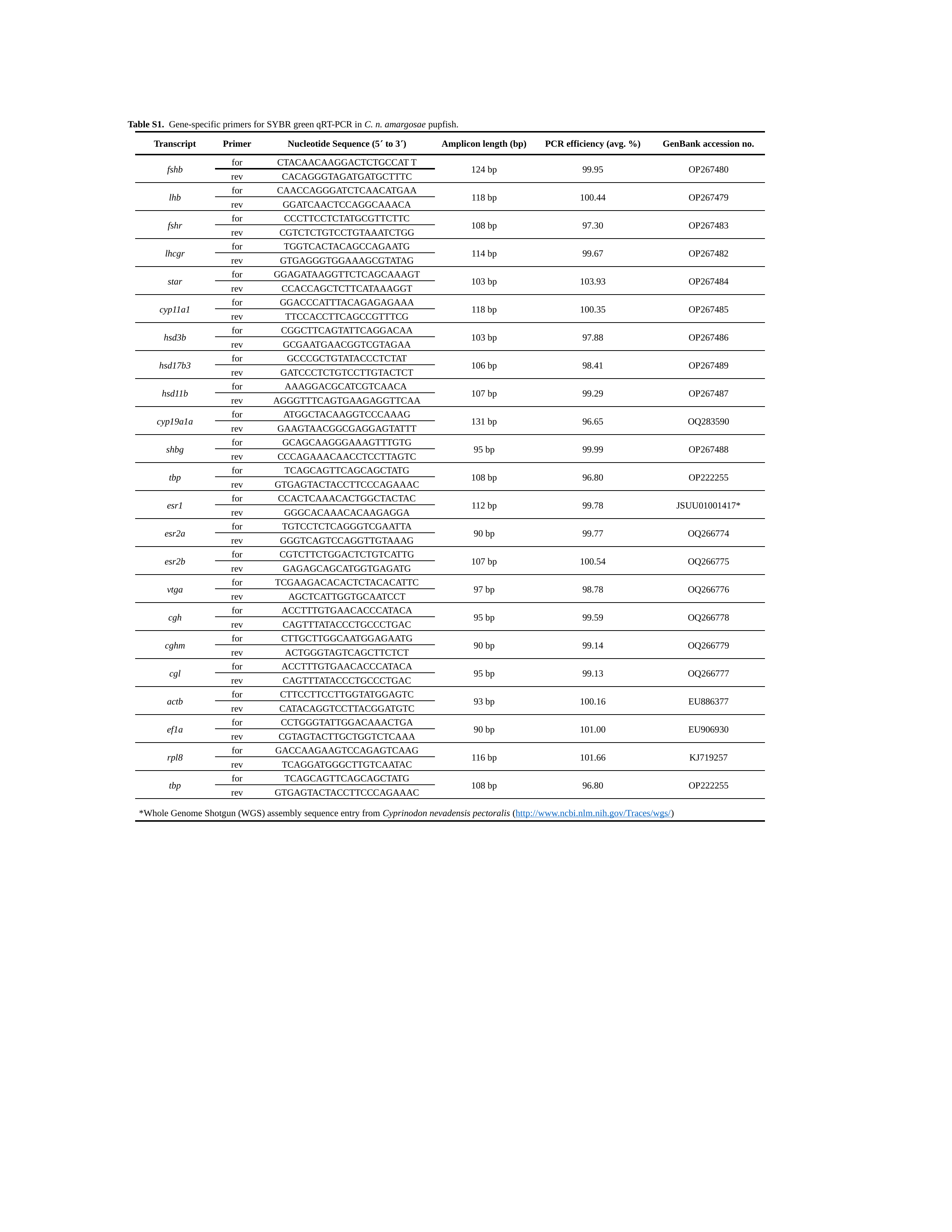

Table S1. Gene-specific primers for SYBR green qRT-PCR in C. n. amargosae pupfish.
| Transcript | Primer | Nucleotide Sequence (5′ to 3′) | Amplicon length (bp) | PCR efficiency (avg. %) | GenBank accession no. |
| --- | --- | --- | --- | --- | --- |
| fshb | for | CTACAACAAGGACTCTGCCAT T | 124 bp | 99.95 | OP267480 |
| | rev | CACAGGGTAGATGATGCTTTC | | | |
| lhb | for | CAACCAGGGATCTCAACATGAA | 118 bp | 100.44 | OP267479 |
| | rev | GGATCAACTCCAGGCAAACA | | | |
| fshr | for | CCCTTCCTCTATGCGTTCTTC | 108 bp | 97.30 | OP267483 |
| | rev | CGTCTCTGTCCTGTAAATCTGG | | | |
| lhcgr | for | TGGTCACTACAGCCAGAATG | 114 bp | 99.67 | OP267482 |
| | rev | GTGAGGGTGGAAAGCGTATAG | | | |
| star | for | GGAGATAAGGTTCTCAGCAAAGT | 103 bp | 103.93 | OP267484 |
| | rev | CCACCAGCTCTTCATAAAGGT | | | |
| cyp11a1 | for | GGACCCATTTACAGAGAGAAA | 118 bp | 100.35 | OP267485 |
| | rev | TTCCACCTTCAGCCGTTTCG | | | |
| hsd3b | for | CGGCTTCAGTATTCAGGACAA | 103 bp | 97.88 | OP267486 |
| | rev | GCGAATGAACGGTCGTAGAA | | | |
| hsd17b3 | for | GCCCGCTGTATACCCTCTAT | 106 bp | 98.41 | OP267489 |
| | rev | GATCCCTCTGTCCTTGTACTCT | | | |
| hsd11b | for | AAAGGACGCATCGTCAACA | 107 bp | 99.29 | OP267487 |
| | rev | AGGGTTTCAGTGAAGAGGTTCAA | | | |
| cyp19a1a | for | ATGGCTACAAGGTCCCAAAG | 131 bp | 96.65 | OQ283590 |
| | rev | GAAGTAACGGCGAGGAGTATTT | | | |
| shbg | for | GCAGCAAGGGAAAGTTTGTG | 95 bp | 99.99 | OP267488 |
| | rev | CCCAGAAACAACCTCCTTAGTC | | | |
| tbp | for | TCAGCAGTTCAGCAGCTATG | 108 bp | 96.80 | OP222255 |
| | rev | GTGAGTACTACCTTCCCAGAAAC | | | |
| esr1 | for | CCACTCAAACACTGGCTACTAC | 112 bp | 99.78 | JSUU01001417\* |
| | rev | GGGCACAAACACAAGAGGA | | | |
| esr2a | for | TGTCCTCTCAGGGTCGAATTA | 90 bp | 99.77 | OQ266774 |
| | rev | GGGTCAGTCCAGGTTGTAAAG | | | |
| esr2b | for | CGTCTTCTGGACTCTGTCATTG | 107 bp | 100.54 | OQ266775 |
| | rev | GAGAGCAGCATGGTGAGATG | | | |
| vtga | for | TCGAAGACACACTCTACACATTC | 97 bp | 98.78 | OQ266776 |
| | rev | AGCTCATTGGTGCAATCCT | | | |
| cgh | for | ACCTTTGTGAACACCCATACA | 95 bp | 99.59 | OQ266778 |
| | rev | CAGTTTATACCCTGCCCTGAC | | | |
| cghm | for | CTTGCTTGGCAATGGAGAATG | 90 bp | 99.14 | OQ266779 |
| | rev | ACTGGGTAGTCAGCTTCTCT | | | |
| cgl | for | ACCTTTGTGAACACCCATACA | 95 bp | 99.13 | OQ266777 |
| | rev | CAGTTTATACCCTGCCCTGAC | | | |
| actb | for | CTTCCTTCCTTGGTATGGAGTC | 93 bp | 100.16 | EU886377 |
| | rev | CATACAGGTCCTTACGGATGTC | | | |
| ef1a | for | CCTGGGTATTGGACAAACTGA | 90 bp | 101.00 | EU906930 |
| | rev | CGTAGTACTTGCTGGTCTCAAA | | | |
| rpl8 | for | GACCAAGAAGTCCAGAGTCAAG | 116 bp | 101.66 | KJ719257 |
| | rev | TCAGGATGGGCTTGTCAATAC | | | |
| tbp | for | TCAGCAGTTCAGCAGCTATG | 108 bp | 96.80 | OP222255 |
| | rev | GTGAGTACTACCTTCCCAGAAAC | | | |
| \*Whole Genome Shotgun (WGS) assembly sequence entry from Cyprinodon nevadensis pectoralis (http://www.ncbi.nlm.nih.gov/Traces/wgs/) | | | | | |
